# Review of taxonomy, geographic distribution, and paleoenvironments of Azhdarchidae (Pterosauria)

**DOI:** 10.3897/zookeys.432.7913

**Published:** 2014-08-11

**Authors:** Alexander Averianov

**Affiliations:** 1 Zoological Institute of the Russian Academy of Sciences, Universitetskaya nab. 1, 199034 Saint Petersburg, Russia and Department of Sedimentary Geology, Geological Faculty, Saint Petersburg State University, 16 liniya VO 29, 199178 Saint Petersburg, Russia

**Keywords:** Pterosauria, Azhdarchidae, taxonomy, distribution, paleoenvironments, Cretaceous

## Abstract

The taxonomy, geographic distribution, and paleoenvironmental context of azhdarchid pterosaurs are reviewed. All purported pteranodontid, tapejarid, and azhdarchid specimens from the Cenomanian Kem Kem beds of Morocco are referred to a single azhdarchid taxon, *Alanqa saharica*. The four proposed autapomorphies of *Eurazhdarcho langendorfensis* from the lower Maastrichtian Sebeş Formation of Romania are based on misinterpretations of material and this taxon is likely a subjective junior synonym of *Hatzegopteryx thambema*. Among 54 currently reported azhdarchid occurrences (51 skeletal remains and 3 tracks) 13% are from lacustrine deposits, 17% from fluvial plain deposits, 17% from coastal plain deposits, 18% from estuarine and lagoonal deposits, and 35% from costal marine deposits. Azhdarchids likely inhabited a variety of environments, but were abundant near large lakes and rivers and most common in nearshore marine paleoenvironments.

## Introduction

Azhdarchid pterosaurs (Azhdarchidae) are the most derived, successful and stratigraphically youngest group of pterosaurs and flourished during the Late Cretaceous after the decline of most other pterosaur groups (Witton 2007; Witton and Naish 2008). The toothed pterodactyloids (Ornithocheiridae) dominated during the Early Cretaceous and earliest Late Cretaceous (Cenomanian and early Turonian). Starting in the late Turonian all pterodactyloids were toothless: Pteranodontidae and Nyctosauridae in the Western Hemisphere and Azhdarchidae worldwide. This shift in dominance from toothed to toothless pterodactyloids apparently reflects some fundamental changes in Cretaceous ecosystems, which we still poorly understand.

The fossil record of pterosaurs is patchy and confined mostly to Konservat-Lagerstätten where exceptional depositional conditions facilitated preservation of fragile pterosaur bones (Butler et al. 2013). Unfortunately, such Lagerstätten are very rare for the Late Cretaceous when most of the evolutionary history of Azhdarchidae took place. Azhdarchidae currently represents a real nightmare for pterosaur taxonomists: most taxa are known from few fragmentary bones, which often do not overlap between named taxa, the few articulated skeletons are poorly preserved (*Zhejiangopterus*), and the best available postcranial material (*Quetzalcoatlus*) has remained undescribed for forty years. Nevertheless, the number of azhdarchid localities is impressive and undoubtedly reflect the important role that these pterosaurs played in the Late Cretaceous ecosystems.

The imperfect nature of the azhdarchid fossil record poses a problem for the taxonomic attribution of their isolated bones. It is a common practice to confine azhdarchid taxa to few diagnostic bones whereas other bones in the locality are classified as Azhdarchidae indet. (Ősi et al. 2005, 2011; Ibrahim et al. 2010). This superficially objective approach actually creates two taxa for the locality, a named taxon and a taxon left in open nomenclature (Azhdarchidae indet.). This approach violates Ockham’s Razor, the principle of parsimony: entities must not be multiplied beyond necessity. In this particular case, the number of azhdarchid taxa in a given locality must not be multiplied *unless* it can be demonstrated by homologous skeletal elements with different structure. Another aspect of this problem is the creation of multiple named, presumably valid, closely related azhdarchid taxa based on materials from the same stratigraphic unit and the same or neighboring localities. For azhdarchids, this practice was introduced by Langston (1981: 102) who referred the smaller specimens of Texas pterosaur to *Quetzalcoatlus* sp. “in the absence of proof that it was the young of the species *northropi*.” Kellner and Langston (1996: 222) were “convinced that the small individuals [*Quetzalcoatlus* sp.] most likely represent a different taxon,” but arguments supporting this view have not been presented. This is wrong and misleading. Ontogenetic, sexual, and individual variation is to be expected in the population whereas the existence of closely related species in the same ecosystem is uncommon. Variability is the null hypothesis and taxonomic distinction should only be hypothesized if the size and morphological variation cannot be accounted for by ontogeny, sexual dimorphism, or allometric scaling. Ignoring of this principle led to unjustified taxonomic inflation and unfounded hypotheses on taxic diversity, niche partitioning, and other aspects of azhdarchid evolutionary history (Butler et al. 2013; Vremir et al. 2013).

This paper provides a review of the taxonomy and distribution of Azhdarchidae based on the principles outlined above. The revised and annotated list of azhdarchid localities is used to assess the preferred paleoenvironments of azhdarchid pterosaurs.

### Institute abbreviations

BMR Burpee Museum of Natural History, Rockford, Illinois, USA.

BSP Bayerische Staatssammlung für Paläontologie und Geologie, Munich, Germany.

CAD Department of Paleontology and Stratigraphy, Jilin University, Changchun, China.

CMN Canadian Museum of Nature, Ottawa, Canada.

CCMGE Chernyshev’s Central Museum of Geological Exploration, Saint Petersburg, Russia.

EME Transylvanian Museum Society, Cluj-Napoca, Romania.

FGGUB Faculty of Geology and Geophysics, University of Bucharest, Bucharest, Romania.

FSAC-KK Faculté des Sciences Ain Chock, Université Hassan II, Casablanca, Morocco.

GMN Geological Museum, Nanjing, China.

HGM Henan Geological Museum, Zhengzhou, China.

HMG Hobetsu Museum, Hobetsu, Japan.

IVPP Institute of Vertebrate Paleontology and Paleoanthropology, Beijing, China.

KCM Kumamoto City Museum, Kumamoto, Japan.

LINHM Long Island Natural History Museum, New York, USA.

LPM Liaoning Paleontological Museum, Beipiao, Liaoning, China.

MC Musée de Cruzy, Cruzy, France.

MCNA Museo de Ciencias Naturales de Alava, Vitoria, Spain.

MDM Mifune Dinosaur Museum, Mifune, Japan.

MGUV Museo del Departamento de Geología, Universidad de Valencia, Valencia, Spain.

ME Museé des Dinosaures, Espéraza, France

MNUFRJ Museu Nacional, Universidade Federal do Rio de Janeiro, Rio de Janeiro, Brazil.

MOR Museum of Rockies, Bozeman, Montana, USA.

MPC Mongolian Paleontological Center, Mongolian Academy of Sciences, Ulaanbaatar, Mongolia.

MPV Museo Paleontológico Municipal de Valencia, Valencia, Spain.

MPCN-PV Vertebrate paleontology Collection, Museo Patagónico de Ciencias Naturales, General Roca, Río Negro, Argentina.

MTCO Muzeul Tarii Crisurilor, Oradea, Romania.

MTM Magyar Természettudományi Múzeum, Budapest, Hungary.

NHMUK Natural History Museum, London, United Kingdom.

NJSM New Jersey State Museum, New Jersey, USA.

NZGS New Zealand Geological Survey, Lower Hutt, New Zealand.

OCP DEK/GE Office Cherifien des Phosphates, Service Geologique, Khouribga, Morocco.

PMOL Paleontological Museum of Liaoning, Shenyang, China.

RTMP Royal Tyrell Museum of Paleontology, Drumheller, Canada.

SGU Saratov State University, Saratov, Russia.

SMNK PAL Staatliches Museum für Naturkunde Karlsruhe, Karlsruhe, Germany.

SMP State Museum of Pennsylvania, Harrisburg, Pennsylvania, USA.

SMU Southern Methodist University, Dallas, Texas, USA.

TMM Texas Memorial Museum, University of Texas, Austin, Texas, USA.

UCMP University of California Museum of Paleontology, Berkeley, California, USA.

UJA University of Jordan, Department of Geology, Amman, Jordan.

USNM National Museum of Natural History, Smithsonian Institution, Washington, D.C., USA.

UWPI Paläontologisches Institut der Universität Wien, Vienna, Austria.

VGI Volzhsk Humanitarian Institute, Volzhsk, Russia.

WAM Western Australian Museum, Perth, Australia.

WDC Wyoming Dinosaur Center, Thermopolis, Wyoming, USA.

YPM-PU Yale Peabody Museum of Natural History, former collection of the Princeton University Museum of Natural History, New Haven, USA.

ZIN PH Zoological Institute, Russian Academy of Sciences, Paleoherpetological Collection, Saint Petersburg, Russia.

ZMNH Zhejiang Museum of Natural History, Hangzhou, China.

## Taxonomy of Azhdarchidae

**Figure 1.**
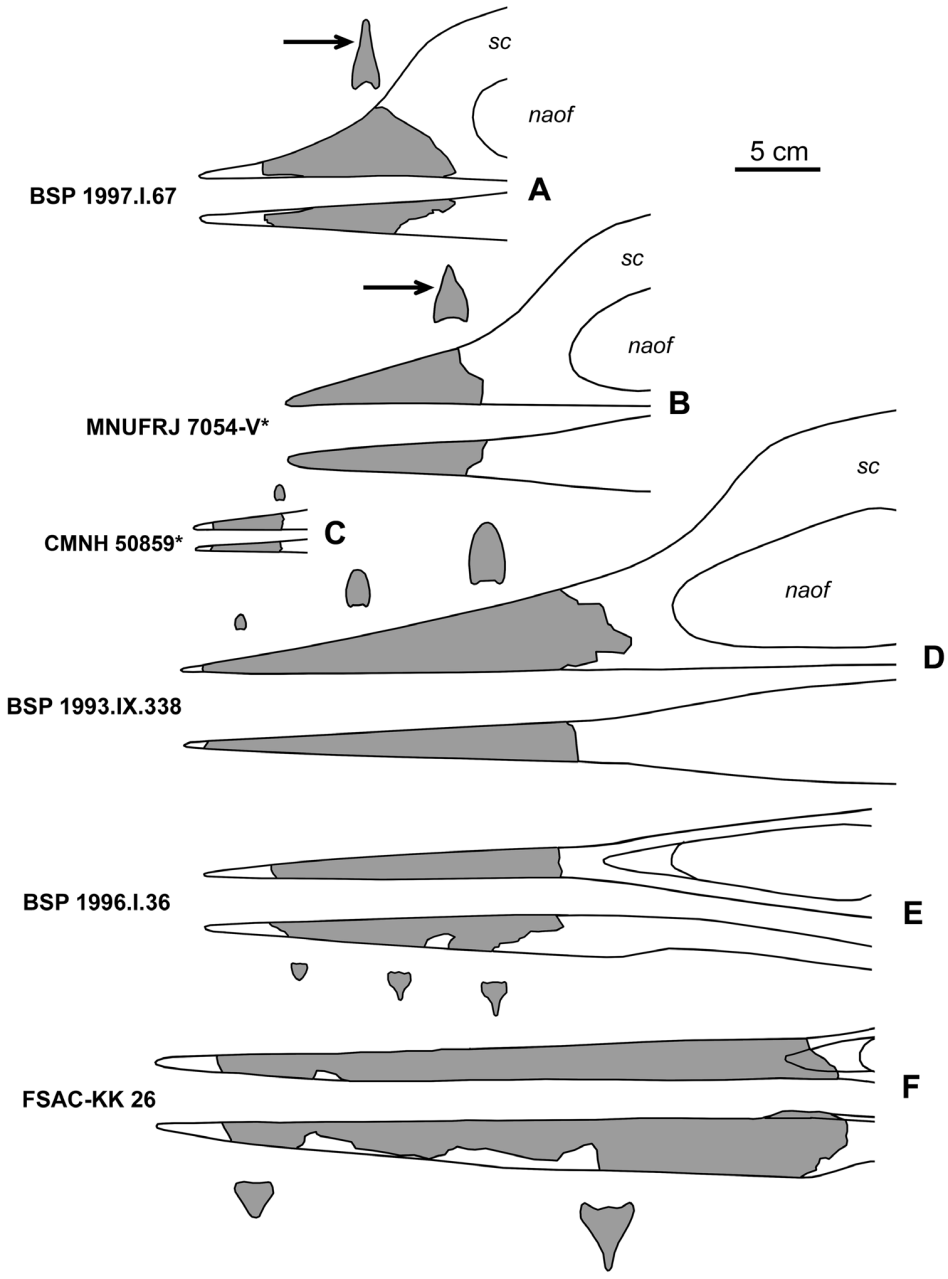
Ontogenetic interpretation of the known jaw fragments of *Alanqa saharica* (all drawn at the same magnification; specimen numbers are shown on the figure; reversed images are marked by asterisk). **A–D** rostrum fragments, in lateral and ventral views **E, F** fragments of mandibular symphysis, in dorsal and lateral views. Figures are modified from Wellnhofer and Buffetaut 1999 (**A, D, E**), Kellner et al. 2007 (**B**), Rodrigues et al. 2011 (**C**), and Ibrahim et al. 2010 (**F**). The arrow indicates the beginning of the sagittal crest on the cross sections of the rostra. *Abbreviations*: *naof* – nasoantorbital fenestra; *sc* – sagittal crest.

***Alanqa saharica*.** I refer to this species all azhdarchid remains from the Cenomanian Kem Kem beds of Morocco, which include edentulous jaw fragments, cervical vertebrae, and a fragmentary humerus (Wellnhofer and Buffetaut 1999; Kellner et al. 2007; Ibrahim et al. 2010; Rodrigues et al. 2011). The jaw fragments show some variation, which was considered taxonomically significant by previous authors who assigned these specimens to three different families: Azhdarchidae, Tapejaridae, and Pteranodontidae. The ontogenetic interpretation of this variation is more parsimonious (Fig. 1). The mandibular fragments could be easily distinguished by their cross-section, where the dorsal convex part is not deeper than half of the dentary depth. The narrow ventral part actually is a mandibular sagittal crest, which does not project beyond the straight ventral border of the dentary in contrast to the dentary sagittal crest in ornithocheirids. Furthermore, on the dorsal surface of the mandibular symphysis, there is a variably developed medial crest whereas the ventral surface of the rostrum is gently concave. Also, the ventral side in lateral profile is less steep compared with the dorsal side of the rostrum (Fig. 1). Among the known fragments of the rostrum, BSP 1997.I.67 represents the earliest ontogenetic stage. Here the rostrum is relatively short and the sagittal crest, poorly differentiated from the rest of premaxilla, begins close to the jaw tip (Fig. 1A). The next stage is represented by MNUFRJ 7054-V, where the rostrum is relatively longer (Fig. 1B). The smallest fragment CMN 50859 most likely represents the tip of the rostrum of an adult or subadult individual, which has the sagittal crest far away from the rostral tip (Fig. 1C). The more complete specimen BSP 1993.I.338 comes from a younger individual because it has a relatively steeper dorsal profile of the rostrum at the anterior end, implying that the entire rostrum was shorter (Fig. 1D). At this stage there is no indication of the sagittal crest in the cross-section of the rostrum. The crest was likely confined to the more posterior part of the skull. The mandibular symphysis BSP 1996.I.36 apparently comes from an individual of a similar ontogenetic age (Fig. 1E). Finally, the largest and presumably oldest specimen in the sample is represented by FSAC-KK 26, the holotype of *Alanqa saharica* (Fig. 1F). It can also not be ruled out that sagittal crest was present only in males.

The known complete cervical vertebrae of *Alanqa saharica* (Rodrigues et al. 2011: figs 2, 4), which are likely IV (LINHM 014) and V (CMN 50801), markedly differ from the cervicals of *Azhdarcho lancicollis* in the unreduced neural spine, which is obviously a primitive feature of the Cenomanian taxon. The humerus of *Alanqa saharica* (Rodrigues et al. 2011: fig. 7) is similar to that of *Azhdarcho lancicollis* except for the distally projecting entepicondyle.

***Azhdarcho lancicollis*.** The nominal genus of Azhdarchidae is known from fragmentary but abundant specimens from the Turonian Bissekty Formation at Dzharakuduk, Kyzylkum Desert, Uzbekistan (Nesov 1984c; Averianov 2010).

***Bakonydraco galaczi*.** This azhdarchid is known from a single locality, Iharkút quarry in Veszprém County, Hungary, in strata of the Santonian Csehbánya Formation (Ősi et al. 2005, 2011). Here I refer to this taxon all pterosaur remains currently known from this locality. In the holotype mandible MTM V2007.110.1 (Ősi et al. 2005: fig. 2), the articular surface for the quadrate is not well preserved but it was likely subdivided into medial and lateral cotyles as in other azhdarchids (contra Ősi et al. 2005). In MTM V2010.98.1, attributed originally to Pterodactyloidea indet., this subdivision is better visible (Ősi et al. 2011: fig. 3A, B). The specimen MTM V2010.99.1, identified as a fourth metacarpal (?) of Pterodactyloidea indet. (Ősi et al. 2011: fig. 3C–E), is a fragment of the distal portion of a femur.

In a recent phylogenetic analysis by Andres and Myers (2013) *Bakonydraco galaczi* clustered in Tapejaridae. Indeed, this taxon is similar to juvenile specimens of *Tapejara wellnhoferi* (SMNK PAL 1137) in the lateral profile of the dentary symphysis, but the older specimens of the latter taxon have prominent ventral dentary crest (AMNH 2440), which has not been reported for *Bakonydraco galaczi*. Furthermore, in the Hungarian taxon, the mandibular symphysis consists of two parts, the beak with its triturating surface, which was possibly covered by a keratinous sheath (as indicated by vascular foramina) and the coalesced dentary rami with a concave dorsal surface (Ősi et al. 2005: fig. 2). In *Tapejara wellnhoferi* the dentary rami are separated posterior to the beak, which is the primitive condition for pterosaurs. The coalesced dentary rami posterior to the beak are present also in *Quetzalcoatlus* (Kellner and Langston 1996: fig. 6) and *Azhdarcho* (unpublished materials), and this character may prove to be a synapomorphy for Azhdarchidae not considered by Andres and Myers (2013). The elongated cervical vertebrae from Iharkút are undoubtedly azhdarchid and the attribution of *Bakonydraco galaczi* to Azhdarchidae seems to be well supported.

***Aralazhdarcho bostobensis*.** The taxon is known from several isolated bones, including atlas-axis and anterior fragment of mid-cervical vertebra, from the Santonian – lower Campanian Bostobe Formation at Shak-Shak in Kyzylorda Province of Kazakhstan (Nesov 1984c; Averianov 2004, 2007b). *Samrukia nessovi* is based on a mandible fragment from the Bostobe Formation of the nearby Akkurgan locality, originally misidentified as a bird (Naish et al. 2012) but reinterpreted as a pterosaur (Buffetaut 2011), is similar to the mandible of *Quetzalcoatlus* (Kellner and Langston 1996: figs 4C, D, 5) in having a peculiar posterolateral process of the lateral cotyle of the mandibular articulation, which could be a synapomorphy for Azhdarchidae. *Samrukia nessovi* is referred here to Azhdarchidae and tentatively considered a subjective junior synonym of *Aralazhdarcho bostobensis*. *Aralazhdarcho bostobensis* is similar to *Quetzalcoatlus* in the structure of posterior part of the mandible (poorly known in other azhdarchid taxa). It differs from all known azhdarchid taxa in the reduction of pneumatic foramina lateral to the neural canal on the cervical vertebrae and the convex rather than saddle-shaped humeral head.

***Volgadraco bogolubovi*.** The species is based on a snout fragment (SGU 46/104a, holotype) and few other isolated bones from the lower Campanian Rybushka Formation at Shirokii Karamysh 2 in Saratov Province, Russia (Averianov et al. 2008). The snout fragment (Averianov et al. 2008: pl. 5, fig. 1) was originally identified as part of the mandibular symphysis, but more likely it represents the rostrum (Novas et al. 2012). In addition to the previously described specimens, a distal syncarpal was recently found at the type locality. This taxon is likely synonymous with *Bogolubovia orientalis*, based on cervical vertebra fragment from the Rybushka Formation at Malaya Serdoba in Penza Province, Russia (Bogolyubov 1914; Nesov and Yarkov 1989). Because the holotype of *Volgadraco bogolubovi* is more diagnostic and that of *Bogolubovia orientalis* is lost will be better to treat the latter taxon as a nomen dubium and refer all azhdarchid bones from Rybushka Formation to *Volgadraco bogolubovi*. Azhdarchid bones are also known from the Rybushka Formation at the Beloe Ozero localitiy in Saratov Province (Averianov et al. 2005; Averianov 2007b, 2008; Averianov and Popov 2014).

***Zhejiangopterus linhaiensis*.** In contrast with most other azhdarchid taxa, this taxon is represented by several articulated skeletons from the Middle Member of the Tangshang Formation (lower Campanian) at Linhai City in Zhejian Province, China (Cai and Wei 1994; Unwin and Lü 1997). The geological age of this unit is 81.5 Ma based on potassium-argon dating (Cai and Wei 1994). The skeletons retain some poorly preserved soft-tissue remains, but the preservation of the bones is rather poor. The holotype (ZMNH M1330) is the skull without cranial crest and apparently belongs to an immature individual.

***Aerotitan sudamericanus*.** It is based on a single rostrum fragment (MPCN-PV 0054) from the Campanian-Maastrichtian Allen Formation at Cerro de Guerra, Río Negro Province, Argentina (Novas et al. 2012). *Aerotitan sudamericanus* was originally distinguished by the proportions of the rostrum and the pattern of neurovascular foramina (Novas et al. 2012). Indeed the anterior rostrum fragment is very narrow transversely, suggesting that the entire rostrum was quite long. This character distinguishes *Aerotitan sudamericanus* from all azhdarchid taxa except *Quetzalcoatlus*, which has a similarly long and narrow rostrum (Kellner and Langston 1996: figs 2, 3). The anterior part of the rostrum in *Quetzalcoatlus* has not been illustrated in ventral view, but the mandibular symphysis (Kellner and Langston 1996: fig. 5C) indicates the narrowness of the rostrum. *Aerotitan* has a single row of neurovascular foramen along the alveolar margin of the rostrum (Novas et al. 2012: fig. 2). In *Bakonydraco*, there are fewer foramina arranged on the lateral surface in two rows, one dorsal to and the other along the alveolar margin (Ősi et al. 2011: fig. 2B). In *Alanqa*, there is a dorsal row and at least one foramen in ventral row (Novas et al. 2012: fig. 3C). The pattern of neurovascular foramina is highly variable in the known rostral fragments of *Azhdarcho lancicollis* (ZIN PH collection). They can be altogether absent, irregularly spaced, or arranged in parallel rows on the palate, form an almost interrupted groove near the alveolar margin on the lateral surface, be situated in the middle of the lateral surface or closer to the dorsal margin, or form two rows on the lateral aspect, one along the alveolar border and another closer to mid-height. ZIN PH 118/44 has a unique pattern of neurovascular foramina: the palatal foramina are slit-like and extend parallel to the alveolar margins anteriorly, but posteriorly the each row is doubled and consists of smaller round foramina; the lateral foramina are very small and sparsely placed very close to the alveolar margin. The larger specimens usually have fewer foramina. This is likely correlated with the slowdown of the growth of the keratinous sheath. According to Novas et al. (2012) in *Quetzalcoatlus* there are no neurovascular foramina on the rostrum. However, this may be due to the poor preservation of the bony surface in described specimens (Kellner and Langston 1996); these foramina are present in all other azhdarchids. *Aerotitan* is very similar to *Quetzalcoatlus* in narrowness of the rostrum and these taxa may be closely related. Except the neurovascular foramina, which are highly variable in azhdarchids, the only significant difference between the two taxa is the convex profile of alveolar border in *Aerotitan*.

***Phosphatodraco mauritanicus*.** The holotype (OCP DEK/GE 111) is a series of closely associated cervicals V-IX and an indeterminate bone from the upper “Couche III” at Site 1 of Sidi Daoui in the Oulad Abdoun Phosphatic Basin, Morocco (Pereda Suberbiola et al. 2003). Kellner (2010: 1076) thought that the "elongated element [on the holotype] that in the original description was regarded as the fifth cervical vertebra is actually formed by two cervical elements, the first being the third and the second the fourth, respectively." If so, the cervicals on the holotype would be III-VIII. This interpretation is improbable and was previously dismissed by Pereda Suberbiola et al. (2003: 81). There are no remnants of zygapophyses (composed of dense bone) in the breakage within the fifth cervical whereas other zygapophyses are well preserved. *Phosphatodraco mauritanicus* is distinct in having relatively long cervical VIII with high neural spine restricted to the posterior part of the vertebra. In *Quetzalcoatlus* cervical VIII has very similar neural spine, but the centrum is much shorter. In *Azhdarcho* cervical VIII is intermediate in length between that of *Phosphatodraco mauritanicus* and *Quetzalcoatlus*. According to the original description, *Phosphatodraco* has no pneumatic canals lateral to the neural canal. However, this could only be established for cervical IX (Pereda Suberbiola et al. 2003: fig. 3e), whereas the anterior end of cervical VI (Pereda Suberbiola et al. 2003: fig. 3f) is poorly preserved and a lateral pneumatic foramen could be present there. The lateral pneumatic foramina are present on cervical IX in *Azhdarcho*, but absent in *Volgadraco* and *Quetzalcoatlus* (Averianov et al. 2008; Averianov 2010).

***Arambourgiania philadelphiae*.** A giant pterosaur known from a mid-cervical vertebra and wing phalanx fragments from the Maastrichtian phosphorites of the Balqa Group at Ruseifa near Amman, Jordan (Frey and Martill 1996; Steel et al. 1997; Martill et al. 1998). The holotype vertebra (UJA VF-1) was originally misinterpreted as a wing metacarpal (Arambourg 1954, 1959) and reinterpreted as a cervical by Lawson (1975b). The original generic name of this largest flying creature, *Titanopteryx* Arambourg, 1959, turned out to be preoccupied ironically by a name for one of the smallest flying animal, a black fly (Simulidae), and was replaced by *Arambourgiania* (Nesov and Yarkov 1989). The holotype of *Arambourgiania philadelphiae* is distinct in being oval in cross-section, with the cotylar and condylar articular surfaces of the centrum higher than wide, and having vertically oriented postexapophyses. The mid-cervical has a costoventral sulcus (Martill et al. 1998: fig. 7b; contra Pereda Suberbiola et al. 2003).

Lewy et al. (1992) reported on two natural endocranial casts, one of which is perfectly preserved, from the upper part of the Phosphorite Unit of the Phosphate Member of the uppermost Mishash Formation (uppermost Campanian) at Oron in southern Israel, which they referred to *Titanopteryx* sp. Although pterosaur nature of the figured endocast (Lewy et al. 1992: pl.1, figs 5, 6) cannot be ruled out (see, for comparison, Witmer et al. 2003), its small size (length around 3 cm) and reported bird-like structure suggest avian affinities.

***Quetzalcoatlus northropi*.** The taxonomic history of this most widely known azhdarchid pterosaur and one of the most popular extinct animals is very confused. When it discovery was first announced it was simply called the Big Bend pterosaur (Lawson 1975b), referring to its provenance from the Maastrichtian Javelina Formation of Big Bend National Park in Texas, USA. In reply to the critique of the first paper, Lawson (1975a: 677) gave a name to the material and designated a type specimen (TMM 41450-3), an articulated wing, but did not provide a diagnosis of the new taxon, which thus is technically a nomen nudum (Sullivan and Fowler 2011). However, an obvious reference to a previous publication (Lawson 1975b), which contains a discussion of diagnostic characters for this taxon, makes the name available (ICZN 1999: Article 13.1.2). To date the only published formal diagnosis of the taxon was provided by Nesov (1991).

Lawson (1975b: 947) indicated the presence of larger and smaller specimens at localities separated by 40 km, but decided without further discussion that “all can be referred to a single species because of the similarity of their humeri, proximal carpals, and second phalanges.” Kellner and Langston (1996) restricted the name *Quetzalcoatlus northropi* to the holotype, which represents the larger specimen, whereas smaller specimens, which are only half size of the holotype, were referred to a new undescribed species, which they left in open nomenclature as *Quetzalcoatlus* sp.

***Eurazhdarcho langendorfensis*.** The most complete European azhdarchid is known from an incomplete and poorly preserved skeleton (EME VP 312) from the lower Maastrichtian Sebeş Formation at Lancrĕm in Transylvania, Romania (Vremir et al. 2013). The new taxon was diagnosed by four proposed autapomorphies: 1) the length of cervical III is 75% that of cervical IV (vs. about 60% in *Zhejiangopterus* and *Quetzalcoatlus*); 2) well-developed and elongated prezygapophyseal pedicles on cervical vertebrae that enclose an angle of 30° relative to the long axis; 3) well-developed preexapophysis with an anteriorly oriented articular facet, separated from the external prezygapophyseal diapophysis by a deep sulcus; 4) lateral pneumatic foramina small and situated lateroventrally to the neural canal. All of these autapomorphies are problematic. Character 1 is a preservational artifact: in cervical IV, the centrum condyle is missing and this was not taken into account when calculating its total length (Vremir et al. 2013: tab. 1). With the length of the centrum condyle added, the length of cervical III could be around 60% that of cervical IV, as in other azhdarchids. The prezygapophyseal pedicels are well developed and elongated in *Azhdarcho* and other azhdarchids, and *Eurazhdarcho langendorfensis* does not really different from them in this respect. Similarly, the preexapophysis, the articular surface for the postexapophysis, is well developed in all azhdarchids, and the “deep sulcus” is the vertebrocostal sulcus, which contained the vertebral artery and vein and is present in all azhdarchids (Pereda Suberbiola et al. 2003; Averianov 2010). The description of neural canal, which potentially can be seen only on the preserved anterior end of cervical IV, is dubious. It is said that the neural canal is preserved as “a prominent internal mold positioned at mid-height between the preexapophyses” (Vremir et al. 2013: 7). However, the structure labelled as “neural canal” on the line drawing (Vremir et al. 2013: fig. 6c) is actually the hypapophysis of the cotylar articular surface of the centrum, as is clearly seen in the photograph (Vremir et al. 2013: fig. 8b). In a better preserved cervical III from Râpa Roşie (Vremir 2010: fig. 16), the lateral pneumatic foramina are small and positioned lateral to the larger neural canal, as in most azhdarchids. This is interesting because *Arambourgiania* is unique among azhdarchids in having the lateral pneumatic foramina larger than neural canal (Martill et al. 1998: figs 5a, 6a, 7a). This argues against the synonymy of *Arambourgiania* and *Hatzegopteryx* proposed by Witton et al. (2010). The main problem of *Eurazhdarcho langendorfensis* is that it cannot be differentiated from *Hatzegopteryx thambema* by size-independent characters. Vremir et al. (2013) acknowledged that known materials on *Eurazhdarcho langendorfensis* and *Hatzegopteryx thambema* have no overlapping elements. Identification of EME VP 312 as an immature specimen of *Hatzegopteryx thambema* remains a distinct possibility once this pterosaur becomes better known.

***Hatzegopteryx thambema*.** The holotype of this giant pterosaur consists of associated parts of the skull (occiput and quadrate condyle with adjacent bones) and a fragment of the proximal portion of a humerus (FGGUB R1083) from the upper Maastrichtian Densuş-Ciula Formation at Vǎlioara in the Haţeg basin, Transylvania, Romania (Buffetaut and Ouaja 2002, 2003). Also a diaphysis of a very large femur (FGGUB R1625, preserved length 385 mm) from the same formation at the nearby locality Tuştea was originally referred to this taxon. Vremir et al. (2013) mentioned an anterior fragment of the mandibular symphysis of a large pterosaur from the type locality attributable to this taxon, as well as six specimens from other localities within Haţeg basin, which may belong to smaller individuals. Skeletal fragments of a very large azhdarchid, including a very large cervical III, are also known from the lower Maastrichtian Bozeş and Sebeş formations in the Transylvanian basin of Romania (Vremir 2010; Vremir et al. 2013).

*Hatzegopteryx thambema* was originally diagnosed by the structure of the quadrate condyle, which is “massive, with smoothly rounded rather than angular condyles, and no notch posterior to the lateral condyle” (Buffetaut and Ouaja 2002: 181). At that time the quadrate condyle was known only for *Quetzalcoatlus* (Kellner and Langston 1996). The construction of the quadrate condyle was interpreted as “helical,” but, as was noted by Averianov (2010: 268), in *Hatzegopteryx thambema* the lateral and medial condyles are separated by a groove, whereas in taxa with the helical craniomandibular joint (*Pteranodon*, *Azhdarcho*, *Quetzalcoatlus*) the condyles are separated by a ridge. In pterodactyloids with long skulls (*Pteranodon*, *Quetzalcoatlus*), the axis of the craniomandibular joint is perpendicular to the longitudinal axis of the skull and the jugal arches are parallel. If the craniomandibular joint is oriented perpendicular to the long axis in *Hatzegopteryx thambema*, the angle between the jugal arches and the transverse axis of the skull is only ~62° (~90° in *Pteranodon*), which suggests a very short skull, certainly less than estimated length of 2.5–3 m (Buffetaut and Ouaja 2002, 2003; Witton 2013).

### Outgroup taxa and their paleoenvironments

Several conflicting phylogenetic hypothesis place different pterodactyloid taxa as the closest relatives of Azhdarchidae (Table 1). In this section, I review all possible azhdarchid outgroup taxa, including non-azhdarchid azhdarchoids, and their paleoenvironments.

**Table 1.** Sister group for Azhdarchidae in the analyses which recover the monophyletic Azhdarchidae.

Sister group	Reference
*Tapejara* + *Tupuxuara* or Tapejaridae + Thalassodromidae	Kellner 1995, 2003, 2004; Wang et al. 2009)
*Tupuxuara* or Thalassodromidae	Unwin 2003; Martill and Naish 2006; Witton 2013
Chaoyangopteridae	Andres and Ji 2008; Lü et al. 2008; Andres and Myers 2013
Thalassodromidae + (Chaoyangopteridae + Tapejaridae)	Pinheiro et al. 2011

***Chaoyangopterus zhangi*.** The holotype (IVPP V13397) and a referred specimen (LPM R0076) are known from two different localities in the Jiufotang Formation of Dapingfang, Chaoyang, Liaoning Province, China (Wang and Zhou 2003; Zhou 2010). The holotype is a subadult (wing span 1.85 m) and the referred specimen is immature (wing span 1.45 m). I refer to this species also HGM 41HIII-305A from an unspecified locality in the same unit in Chaoyang County, the holotype of *Shenzhoupterus chaoyangensis* (Lü et al. 2008). The latter specimen is also immature (wing span 1.40 m), with the scapulacoracoid and extensor tendon process still unfused. The syncarpals "appear to be coossified" according to Lü et al. (2008: 892) but this claim is not supported by the description or illustration of the bones. The two characters, the unusually slender premaxillary bar bounding the nasoantorbital opening and the extension of the nasoantorbital opening posterior to the jaw joint, comprising the diagnosis of Chaoyangopteridae by Lü et al. (2008), are based solely on this specimen and cannot be observed in any other specimen referred to this family. The first character is likely a juvenile trait of HGM 41HIII-305A. The second character is possibly based on misinterpretation of the specimen. The skull might not be complete posteriorly and the posteroventral corner of the nasoantorbital fenestra is likely filled by cranial and possibly some postcranial bones.

The Aptian Jiufotang Formation is the uppermost formation of the Jehol Group, an important Konservat-Lagerstätte producing numerous, often articulated specimens of diverse plants, invertebrates, and vertebrates (Zhou et al. 2003; Chang et al. 2008; Selden and Nudds 2012). The Jiufotang Formation consists of predominantly lacustrine deposits: sandstones, shales, and mudstones with intercalated tuffs. The volcanic activity was relatively weak compared to that recorded by the underlying Yixian Formation. The biota from the Jiufotang Formation is characterized by a distinct complex of freshwater fishes (*Jinanichthys* ichthyofauna) and by the abundance of ornithurine birds (*Cathayornis*-*Chaoyangia* aviafauna) (Chang et al. 2008). The paleoenvironment was dominated by wetlands and lakes.

***Jidapterus edentus.*** This pterosaur is known from two specimens, the immature CAD 01, the holotype of *Jidapterus edentus* (Dong et al. 2003) and the adult GMN 03-11-002, the holotype of *Eoazhdarcho liaoxiensis* (Lü and Ji 2005), both from the Jiufotang Formation in Chaoyang County, Liaoning Province, China. In the data matrix by Andres and Ji (2008) the two taxa have 30 identical codings (27% of 111 characters) and differ only in the coding of two characters. In *Jidapterus*, one of the metacarpals (metacarpal III according to Andres and Myers (2013) or metacarpal I according to Lü et al. (2008)) articulates with the carpus, whereas in *Eoazhdarcho* metacarpals I-III do not articulate with carpus. The second character is the length of wing phalanx 2 relative to the length of wing phalanx 1, which was miscalculated for *Eoazhdarcho*. Actually this ratio is 0.78 (Lü and Ji 2005), nearly the same as in *Jidapterus* (0.71; Dong et al. 2003) and both taxa should be coded for the same character state. Wang et al. (2005) previously suggested that *Jidapterus* might be congeneric or even conspecific with *Chaoyangopterus*. The two taxa can be differentiated by the dorsal profile of the skull, which is straight in the former and distinctly concave in the latter. *Jidapterus* might also have a relatively longer rostrum and mandibular symphysis, but the skulls are insufficiently known in these taxa.

***Tupuxuara longicristatus.*** Here I accept the synonymy of *Tupuxuara longicristatus*, *Tupuxuara leonardi*, and *Thalassodromeus sethi* proposed by Martill and Naish (2006). *Tupuxuara deliradamus*, described by Witton (2009), is also added to this list of synonyms. *Tupuxuara longicristatus* is known from several specimens, including mostly undescribed skeletons and skulls, from the Albian Romualdo Formation in Ceará, Brazil (Kellner and Hasegawa 1993; Kellner and Campos 1994, 2002, 2007; Kellner 2004; Veldmeijer et al. 2005; Martill and Naish 2006; Martill and Witton 2008; Witton 2009; Aires et al. 2014). The two cervical vertebrae from the Romualdo Formation, GIUA 4895, the "paratype" of *Santanadactylus brasilensis* (Buisonje 1980), likely belong to this species as well. *Santanadactylus spixi* from the Romualdo Formation (Wellnhofer 1985) is a nomen dubium probably based on postcranial remains of *Tupuxuara longicristatus*.

The Albian Romualdo Formation, formerly known as Santana Formation, is another important Konservat-Lagerstätte with excellently, usually three-dimensionally preserved fossils of plants, invertebrates, and vertebrates (Maisey 1991; Martill 1993; Selden and Nudds 2012). The Romualdo Formation consists of non-fluvial silts and sands and a series of laminated shales with fossil-bearing concretions. The Romualdo Formation was deposited under brackish-water conditions. It represents either a lagoonal setting or a basin with only restricted connections to waters of normal marine salinity (Maisey 1991; Martill 1993; Naish et al. 2004b). There are several mass-mortality horizons with numerous fish fossils. Terrestrial vertebrates are rare and were introduced from nearby shoreline environments. The climate was arid or semi-arid. The flora consists largely of plants of a xerophytic nature. It is dominated by the gymnosperm *Brachyphyllum*, which had succulent leaves, and cycadophytes.

***Tupuxuara* sp.** An undescribed taxon, similar to *Tupuxuara longicristatus* is known from the Aptian Crato Formation in Ceará State, Brazil. It is represented by a complete skeleton with skull in a private collection and several incomplete postcranial skeletons (Martill and Frey 1998a, 1999; Sayão and Kellner 1998, 2007; Unwin and Martill 2007). For the paleoenvironment of the Crato Formation see locality 1 in the next section.

**Cornet azhdarchoid.** Dyke et al. (2011) identified MTCO 21269, a fragment of the anterior end of an elongated cervical vertebra, from the Berriasian-Valanginian Lower Bauxite Formation at Cornet, Romania, as a possible azhdarchid. The complete length of the vertebra is unknown, but its reduced neural spine and merged vertebral centrum and neural arch suggested affinities with Azhdarchidae. The rostrum fragment MTCO 18262 from the same locality (Benton et al. 1997: fig. 12E; Dyke et al. 2011: figs 7G, H, 8N, O) was referred to Dsungaripteridae because of the alveoli being confined to the posterior part of the rostrum. However, interpretation of these openings as neurovascular foramina rather than alveoli is equally possible and, if correct, this specimen may belong to Azhdarchoidea as well. There are some other pterosaur bones from the Cornet site that may belong to Azhdarchoidea (MTCO 17738 and 17755, fragments of proximal portions of humeri and MTCO 17642, fragment of a distal portion of a humerus).

The Cornet locality documents an insular fauna inhabiting one of small islands along the northern shores of Tethys (Benton et al. 1997). The fossils were washed into deep fissures and caves formed within a karst of the latest Jurassic marine limestones. Charophytes, ostracods, and freshwater gastropods indicate a lacustrine paleoenvironment (Benton et al. 1997). The humid tropical climate favored the formation of bauxites. The vertebrate fauna includes abundant small ornithopods, rare ankylosaurs and non-avian theropods, birds and pterosaurs (Bock and Buehler 1996; Benton et al. 1997; Posmosanu and Cook 2000; Galton 2009; Dyke et al. 2011).

***Palaeornis cliftii*.** This taxon should be considered a nomen dubium and is based on a humerus preserved as two fragments (NHMUK 2353 and 2353a) from the Valanginian Hastings Beds Group of the Weald Sub-basin (probably from the Upper Tunbridge Wells Formation) at the historical Tilgate Forest locality near Cuckfield in England (Witton et al. 2009). The specimen was referred to Lonchodectidae by Witton et al. (2009) based on comparison with the humeri from the Cambridge Greensand of England and alternatively referred to the toothless azhdarchoid *Ornithostoma sedgwicki* (Averianov 2012). The Hastings Beds have produced a rich vertebrate fauna, including fishes, turtles, plesiosaurs, crocodyliforms, dinosaurs, and mammals (Benton and Spencer 1995), which likely inhabited a coastal marine environment.

**Wessex azhdarchoid.** This pterosaur is known from a complete humerus in a private collection and discovered in variegated mudstones of the Barremian Wessex Formation of the Isle of Wight, England (Witton et al. 2009). The Wessex Formation represents a mixture of fluvial, floodplain, and lacustrine environments on a nearshore alluvial plain (Insole and Hutt 1994). The diverse vertebrate fauna from Wessex Formation includes hybodontiform and carcharhiniform sharks, osteichthyans, albanerpetontids, salamanders, frogs, lepidosaurs, pterosaurs, ankylosaurs, ornithopods, pachycephalosaurs, sauropods, non-avian theropods, and mammals (Blows 1995; Hutt et al. 2001; Martill and Naish 2001; Naish and Martill 2002; Evans et al. 2004; Naish et al. 2004a; Sweetman 2004, 2006, 2008, 2009; Weishampel et al. 2004; Sweetman and Underwood 2006; Benson et al. 2009; Mannion 2009; Sweetman and Martill 2010; Mannion et al. 2011; Sweetman and Gardner 2013).

***Vectidraco daisymorrisae*.** This taxon is based on a partial skeleton (NHMUK PV R36621; pelvis and associated vertebrae) apparently from the lower Aptian Chale Clay member of the Atherfield Formation of the Isle of Wight, England (Naish et al. 2013).

The Chale Clay Member (Atherfield Clay) is composed of silty clay with numerous small clay-ironstone nodules and accumulated in shallow marine conditions with storm events resulting in silty lags (Simpson 1985). It contains pyritized wood, remains of bivalves and rare ammonites, teeth of hybodontiform sharks and pterosaurs (Benton and Spencer 1995; Naish et al. 2013).

***Ornithostoma sedgwicki*.** This taxon, reviewed by Averianov (2012), includes several edentulous jaw fragments, skull fragment, and some postcranial elements from the Upper Greensand Formation or Cambridge Greensand Member of the Lower Chalk Formation in Cambridgeshire, England (exact stratigraphic provenance for the most specimens is unknown). The fossils from both these stratigraphic units are phosphatized and partially reworked from the late Albian Gault Formation (Unwin 2001; Fischer et al. 2014). The Cambridge Greensand vertebrate assemblage includes diverse fishes, marine reptiles, birds, pterosaurs, and rare dinosaurs (Seeley 1870; Lydekker 1888; Hooley 1914; Collins 1970; Elzanowski and Galton 1991; Unwin 2001; Barrett and Evans 2002; Galton and Martin 2002; Martill and Unwin 2012; Rodrigues and Kellner 2013; Fischer et al. 2014). The abundance of phosphatized bones of diverse marine and terrestrial vertebrates likely reflects a shallow-water ecosystem based on planktonic organisms flourishing in upwelling waters rich in phosphorus and other minerals.

**Grandpré azhdarchoid.** A mid-cervical vertebra identical to those of *Ornithostoma sedgwicki* has been reported from the Albian Greensand (Sables verts) at Grandpré in the Ardennes, France (Buffetaut 2012). It was identified originally as Azhdarchidae indet. but cannot be referred to Azhdarchidae because of its unreduced neural spine. The Sables verts with phosphate nodules are comparable to the Cambridge Greensand in England. They were similarly deposited in the shallow basin with intensive upwelling. This phosphorite horizon can be traced further to the east in Poland and western Russia (Averianov 2004; Popov and Machalski 2014).

***Bennettazhia oregonensis*.** A humerus and two fused dorsal vertebrae (USNM 11925) from the Albian Hudspeth Formation at Nelson Creek, Oregon, USA, was originally described as *Pteranodon* (?) *oregonensis* (Gilmore 1928). Bennett (1989) first considered this specimen a possible azhdarchid, but later noted its similarity to Dsungaripteridae (Bennett 1994). Nesov (1991) erected a new genus *Bennettazhia* within Azhdarchidae for this species. It does not appear referable to Dsungaripteridae because of the thin bony walls of the humerus (Habib 2007). The humerus of USNM 11925 agrees well with the morphology of this bone in *Azhdarcho lancicollis*, except for the distally somewhat expanded deltopectoral crest and unusual shape of humerus in distal view, which is however poorly preserved in USNM 11925 (Averianov 2010). The two fused dorsals of USNM 11925 are possible not part of the notarium or synsacrum (Averianov 2010); the fusion of free dorsals may occur in old individuals. *Bennettazhia oregonensis* is best considered a non-azhdarchid azhdarchoid.

The Hudspeth Formation consists of thick sequences of hemipelagic mudstone that contain subordinate siltstones and thin beds of turbiditic sandstone (Dorsey and Lenegan 2007). The fauna consists mostly of marine invertebrates (Vega et al. 2010). The holotype of *Bennettazhia oregonensis* and an ichthyosaur centrum, found together with ammonites, are only vertebrate remains reported from this stratigraphic unit (Gilmore 1928; Merriam and Gilmore 1928).

***Radiodactylus langstoni*.** An isolated humerus (SMU 72547) from the upper Aptian – lower Albian Glen Rose Formation at Squaw Creek, Texas, USA, was initially attributed to Azhdarchidae indet. (Murry et al. 1991) but later referred to a new taxon, *Radiodactylus langstoni* (Andres and Myers 2013). According to Andres and Myers (2013), the capitulum (=ventral condyle) is damaged and missing some of its articular surface. But it seems more likely that only small portion of posterodorsal articular surface is missing, along with most of the ectepicondyle. SMU 72547 is unusual and distinctly different from the humeri of *Azhdarcho lancicollis* and *Bennettazhia oregonensis* in the nearly round shape of the humerus in distal view. The capitulum has almost the same width as the trochlea (=dorsal condyle), whereas in *Azhdarcho lancicollis* it is about twice as wide (Averianov 2010: fig. 24C). A small pneumatic foramen on the distal surface of the humerus in SMU 72547 (Murry et al. 1991; Andres and Myers 2013) is certainly not compatible with the hypertrophied distal pneumatic foramen on the humerus in ornithocheirids and istiodactylids. In *Azhdarcho lancicollis* some small foramina or pneumatic fenestrae may be also present in this region (Averianov 2010). The distal end of the humerus in “Tapejaridae” (Rodrigues et al. 2011: fig. 8B) is very similar to that of *Azhdarcho lancicollis*, with the capitulum distinctly wider than the trochlea, the pneumatic foramen absent, and the ulnar tuberculum present (absent in SMU 72547). The phylogenetic position of *Radiodactylus langstoni* as a non-azhdarchid azhdarchoid (Andres and Myers 2013) seems well corroborated.

The Glen Rose Formation is composed of nearshore marine limestone with abundant marine invertebrates, pollen, and fossil wood, and represents a late Aptian to early Albian marine transgression in the region (Behrens 1965; Davis 1974; Perkins 1974; Jacobs and Winkler 1998). The Glen Rose Formation is famous for its dinosaur footprints (Jacobs 1995). The vertebrate fauna documented by skeletal remains includes diverse chondrichthyan and osteichthyan fishes, amphibians, crocodyliforms, and sauropods (Langston 1974; Winkler et al. 1990; Barck 1992; Welton and Farish 1993; Rogers 2000, 2003; Tidwell and Carpenter 2003). The pterosaur assemblage consists of *Radiodactylus langstoni* and the ornithocheirid *Coloborhynchus wadleighi* (Lee 1994; Andres and Myers 2013).

***Cretornis hlavaci*.** This taxon is represented by a fragmentary wing skeleton from the middle-upper Turonian Jizera Formation (=Iser-Schichten) in the Czech Republic (Fritsch 1881a, b). The species epithet is often misspelled as *hlavatschi* following unjustified modification by Lydekker (1888). Jianu et al. (1997) have studied the holotype of *Cretornis hlavaci* and found that it “clearly pteranodontid based on having a caudally directed ulnar crest, a warped deltopectoral crest, and a triangular cross-section of the distal end.” All these claims are incorrect. In azhdarchoids, the ulnar crest is directed caudally (ventrally in flight position), similar to the condition in ornithocheirids and pteranodontids. Bennett (1989: fig.2(6, 7)) introduced this character based on comparison with the humerus USNM 11925 (holotype of *Bennettazhia oregonensis*), where the ulnar crest is almost totally missing. On the humerus of *Cretornis hlavaci* the deltopectoral crest of is not “warped” and its distal end is not triangular in distal view (see description and discussion below). *Cretornis hlavaci* is a valid taxon of azhdarchoid pterosaurs. A redescription is currently in preparation by Averianov and Ekrt.

The Jizera Formation is composed mostly of marlstones and siliciclastic sandstones and dated as middle-late Turonian. It is underlying by the lower Turonian Bílá Hora Formation and overlying by the upper Turonian – Coniacian Teplice Formation (Uličný 2001; Wiese et al. 2004). The Jizera Formation is part of a transgressive-regressive cycle and was deposited during the regressive phase after the maximum transgression characterized by the Bílá Hora Formation, which is composed of marlstones and micritic limestones. The vertebrate fauna of Jizera Formation, except the pterosaur *Cretornis hlavaci*, consist of diverse chondrichthyan and osteichthyan fishes and marine reptiles (Fritsch 1883; Ekrt et al. 2008; Kear et al. 2014).

***Montanazhdarcho minor*.** The holotype (MOR 691) is a partial skeleton from an unspecified locality within the upper Campanian Two Medicine Formation in Glacier County, Montana, USA (Padian et al. 1995; McGowen et al. 2002). Originally this taxon was referred to Azhdarchidae but its short wing metacarpal, which is only 89% of the ulna length, and the pneumatic foramen on the distal portion of the radius suggest that it is a non-azhdarchid azhdarchoid. A redescription of this specimen will be published elsewhere. For the paleoenvironment of the Two Medicine Formation see locality 30 in the next section.

## Review of localities of skeletal fossils of Azhdarchidae

### Locality 1. Unspecified localities within Crato Formation (Fig. 2)

**Geographic position.** Area between Nova Olinda, Santana do Cariri and Tatajuba, in southern Ceará, Brazil.

**Stratigraphy.** Nova Olinda Member of Crato Formation.

**Age.** Late Aptian – early Albian (Batten 2007).

**Depositional environment.** Laminated micritic limestone deposited in lower energy waters. Originally the environment of deposition was considered lacustrine, but, based to more reliable data, much of the formation was deposited under saline conditions with marine waters entering the Crato lagoon (Selden and Nudds 2012). Most likely the water was hypersaline due to the arid climate, which is supported by salt pseudomorphs after hopper-faced halite and a thick sequence of evaporates in the overlying Ipubi Formation (Martill et al. 2007; Selden and Nudds 2012). Life at the bottom of the Crato lagoon was prevented by a salinity-stratified water column with hypersaline, oxygen-deficient bottom waters (Selden and Nudds 2012). Thus benthic organisms, except cyanobacterial mats, and signs of bioturbation are lacking in the laminated layers of Crato Formation. These factors also account for the good preservation of the fossils on the lagoon bottom, which often show remains of soft parts, making this unit an important Konservat-Lagerstätte. The fishes from the Crato Formation, notably the most common gonorhynchiform *Dastilbe* (Davis and Martill 1999; Dietze 2007), were likely euryhaline and great numbers of their fossils could be explained by mass mortality caused by influx of marine waters led to sudden increase in salinity (Selden and Nudds 2012). These fish mortality events may have attracted pterosaurs to the Crato lagoon in great numbers. The bodies of terrestrial vertebrates were possibly washed into the Crato lagoon by rivers entering the basin; such finds are exceptionally rare.

**Associated fauna.** Abundant crustaceans, centipedes, arachnids, insects, and fishes; rare frogs; rare pelomedusoid turtles, crocodyliforms, lizards, non-avian dinosaurs, and birds (Evans and Yabumoto 1998; Kellner 2002; Fielding et al. 2005; Martill et al. 2007; Báez et al. 2009; Figueiredo and Kellner 2009; Fortier and Schultz 2009; Simões 2012). The abundance of taxa is inversely related to their degree of terrestriality. The pterosaur assemblage, aside from azhdarchids, includes “tapejarids” and toothed ornithocheirids (Unwin and Martill 2007; Barrett et al. 2008). In the fish assemblage there is a notable presence of marine taxa such as ichthyodectiforms (Leal and Brito 2004).

**Paleoenvironment.** Coastal lagoon. Most pterosaur specimens from the Crato Formation are subadult to adult (Unwin and Martill 2007), which suggests that nesting sites were quite far from this environment.

**Material.** Azhdarchidae indet.: SMNK PAL 3843, articulated scapulacoracoid and humerus. SMNK PAL 2342, partial skeleton including wing metacarpal and wing phalanges 1–3 (estimated wing span ~2.2 m). MNUFRJ 4729-V, partial skeleton including scapulocoracoid and almost complete articulated wing (estimated wingspan ~2 m).

**References.** Martill and Frey 1998a, b; Sayão and Kellner 1998; Martill and Frey 1999; Frey et al. 2003; Unwin and Martill 2007.

**Comments.** SMNK PAL 2342 was referred to Azhdarchidae because of the T-shaped cross-section of the second and third wing phalanges (Martill and Frey 1998a: figs 4, 5; 1999: figs 2, 3). This attribution was questioned by Kellner (2004) and Sayão and Kellner (2007). The latter authors stated that the T-shaped cross-section of the second and third wing phalanges is also found in the “Tapejaridae” from Crato Formation of Brazil and Jiufotang Formation of China. Following this critique, SMNK PAL 2342 was identified as Tapejaridae indet. by Unwin and Martill (2007) and Elgin et al. (2011). The only cited Brazil “tapejarid” with T-shaped wing phalanges is MNUFRJ 4729-V (Sayão and Kellner 1998), a wing skeleton whose attribution to *Tupuxuara* or a related taxon cannot be demonstrated and which is referred here to Azhdarchidae based on this feature. The tubercle on the posteroventral margin of the coracoid, a purported synapomorphy for “Tapejaridae” (Kellner 2004; Aires et al. 2014), has not been mentioned for this specimen (Sayão and Kellner 1998). Concerning the Chinese “tapejarids,” the presence of T-shaped wing phalanges have never been demonstrated for them, and it was explicitly stated that the lack of this feature exclude them from Azhdarchidae (Lü et al. 2006: 321). SMNK 3843 was previously identified as a possible tapejaroid (Frey et al. 2003: fig.1c), non-azhdarchid azhdarchoid (Elgin and Campos 2012), or an azhdarchid (Geist et al. in press). The scapulacoracoid of SMNK PAL 3843 has a coracoid flange rather than a tubercle and is more similar to that of azhdarchids (Frey et al. 2003: fig. 1b; Averianov 2010) than to thalassodromids (Aires et al. 2014: fig.5A–D).

**Figure 2.**
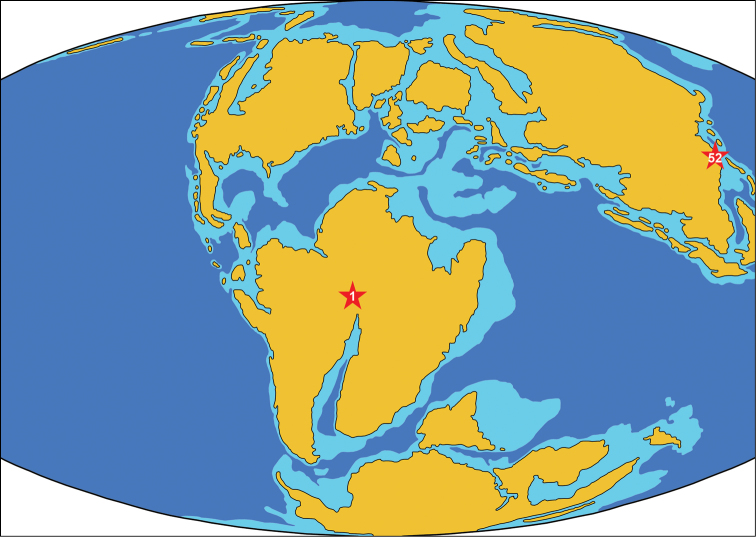
Paleogeographic map of the Early Cretaceous (120 Ma) showing Aptian-age localities of azhdarchids. The numbers of localities correspond to the list in the text. The map is modified from http://jan.ucc.nau.edu/rcb7/120moll.jpg

### Locality 2. Aferdou N'Chaft (Fig. 3)

**Geographic position.** Near Begaa, Province d'Errachidia, Morocco.

**Stratigraphy.** “Continental Intercalaire” (Lavocat 1949). Kem Kem beds (Sereno et al. 1996). Ifezouane Formation (Cavin et al. 2010).

**Age.** Early-middle Cenomanian.

**Depositional environment.** The Kem Kem beds include a lower part consisting of red sandstones with cross-bedded stratifications (Ifezouane Formation) and an upper part with lagoonal gypsiferous marly sandstones and green marls (Aoufous Formation). The latter formation is overlain by limestones of the Akrabou Formation, which record a major late Cenomanian-Turonian transgression. The depositional environment was fluviatile or deltaic for the Ifezouane Formation and lagoonal for the Aoufous Formation (Ettachfini and Andreu 2004; Cavin et al. 2010; Ibrahim et al. 2010). The Aoufous Formation was deposited under hypersaline conditions (Cavin et al. 2010).

The diversity of hybodontiform sharks and presence of two species of marine lamnoid sharks suggests that the fluvial beds of Ifezouane Formation were deposited under brackish-water estuarine rather than freshwater conditions. This is also supported by the abundance of sclerorhynchid teeth. Pristids, the closest modern relatives of sclerorhynchids, are marine, brackish and freshwater (Wueringer et al. 2009).

**Associated fauna.** A rich vertebrate fauna comprising about 80 terrestrial, freshwater and brackish-water taxa is known from Kem Kem beds (Cavin et al. 2010). Most of the vertebrates, including pterosaurs, come from the Ifezouane Formation, while the Aoufous Formation has mainly yielded rostral teeth of the sclerorhynchid *Onchopristis numidus* (Cavin et al. 2010). The vertebrate assemblage from the Ifezouane Formation includes hybodontiforms (*Asteracanthus aegyptiacus*, *Distobatus nutiae*, *Tribodus* sp., *Lissodus* sp.), sclerorhynchiforms (*Onchopristis numidus*, *Marckgrafia lybica*), lamniforms (*Serratolamna amonensis*, Cretoxyrhinidae indet.), lungfishes (two taxa), coelacanths (possibly two taxa), polypterids (several taxa), semionotiforms (at least two taxa), halecomorphs (two taxa), several taxa of teleosteans including the ichthyodectiform *Aidachar pankowskii*, sirenid salamanders, frogs (pipoid and non-pipoid), snakes (several taxa including marine Nigeropheidae), pleurodiran turtles (four families), lizards, diverse crocodyliforms, sauropods, non-avian theropods, and birds (Lavocat 1954; Tabaste 1963; Wenz 1981; Martin 1984; Gmira 1995; Russell 1996; Sereno et al. 1996; Tong and Buffetaut 1996; Forey 1997; Forey and Grande 1998; Dutheil 1999; Larsson and Sidor 1999; Taverne and Maisey 1999; Taverne 2000; Cavin and Brito 2001; Cavin and Forey 2001, 2004, 2008; Buffetaut and Ouaja 2002; Gaffney et al. 2002, 2006; Dal Sasso et al. 2005; Mahler 2005; Forey and Cavin 2007; Larsson and Sues 2007; Rage and Dutheil 2008; Pittet et al. 2009; Sereno and Larsson 2009; Cavin et al. 2010; Forey et al. 2011).

Except for azhdarchid pterosaurs, there are isolated teeth and rostrum fragments of Ornithocheiridae from the Kem Kem beds (Mader and Kellner 1999; Wellnhofer and Buffetaut 1999).

**Paleoenvironment.** Estuarine.

**Material.** *Alanqa saharica*: FSAC-KK 26, mandibular symphysis (holotype). FSAC-KK 27, rostrum. FSAC-KK 34, posterior end of cervical vertebra.

**References.** Ibrahim et al. 2010.

### Locality 3. Boumerade (Fig. 3)

**Geographic position.** Near Maider Lake, Province d'Errachidia, Morocco.

**Stratigraphy.** Ifezouane Formation.

**Age.** Early-middle Cenomanian.

**Depositional environment.** As for locality 2.

**Associated fauna.** As for locality 2.

**Paleoenvironment.** Estuarine.

**Material.** *Alanqa saharica*: FSAC-KK 31, mandibular symphysis.

**References.** Ibrahim et al. 2010.

**Figure 3.**
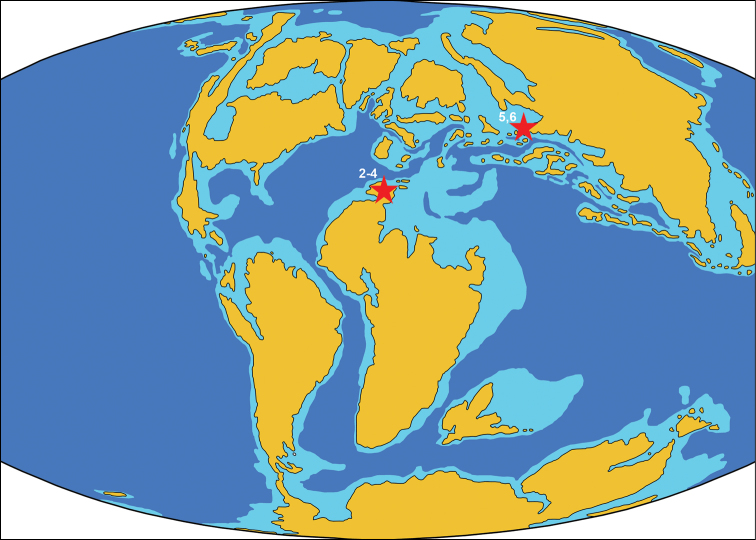
Paleogeographic map of the early Late Cretaceous (105 Ma) showing Cenomanian-age localities of azhdarchids. The numbers of localities correspond to the list in the text. The map is modified from http://jan.ucc.nau.edu/rcb7/105moll.jpg

### Locality 4. Taouz (Fig. 3)

**Geographic position.** Province d'Errachidia, Morocco.

**Stratigraphy.** Ifezouane Formation.

**Age.** Early-middle Cenomanian.

**Depositional environment.** As for locality 2.

**Associated fauna.** As for locality 2.

**Paleoenvironment.** Estuarine.

**Material.** *Alanqa saharica*: east of Taouz city: BSP 1993.IX.338, rostrum; BSP 1997.I.67, rostrum; BSP 1996.I.36, mandibular symphysis.

Region of the Hamada du Guir, Taouz: LINHM 014, cervical vertebra; MNUFRJ 7054-V, rostrum.

Unknown locality: CMN 50859, rostrum; CMN 50801, cervical vertebra; CMN 50814, humerus.

**References.** Kellner and Mader 1996; Wellnhofer and Buffetaut 1999s; Rodrigues et al. 2006, 2011; Kellner et al. 2007; Ibrahim et al. 2010.

### Locality 5. Khodzhakul (Fig. 3)

**Geographic position.** Escarpment north of dried-up lake Khodzhakul, southwestern Kyzylkum Desert, Karakalpakistan, Uzbekistan.

**Stratigraphy.** Lower or middle part of Khodzhakul Formation.

**Age.** Latest Albian(?) or early Cenomanian.

**Depositional environment.** Nearshore marine. The vertebrate assemblage from this locality is different from that of the nearby Sheikhdzheili locality in the same formation in the much greater abundance of marine sharks and greater rarity of terrestrial elements. Nesov (1990a) interpreted this locality as having formed in the channel connecting a marine bay with lagoons and lakes situated behind the beach-ridge. According to Nesov and Mertinene (1986), the great diversity of crustaceans and chondrichthyans and the abundance of bottom dwellers and durophagous forms among fishes indicate deposition in a shallow basin with relatively high salinity. Nesov (1997) thought that the chimaeriforms from Khodzhakul possibly live in brackish waters but their association with other typically marine chondrichthyans and ammonites indicates that the depositional basin had normal marine salinity. The lamniform shark *Hispidaspis gigas* is dominant in the chondrichthyan assemblage, comprising 38.2% of a sample of 3467 teeth (Nesov and Mertinene 1986).

**Associated fauna.** Crustaceans, ammonites (Placenticeratidae), synechodontiform sharks (*Synechodus dispar*, *Paraorthacodus recurvus*), hybodontiform sharks (*Hybodus hodzhakulensis*, *Hybodus nukusensis*, *Polyacrodus* spp., *Acrodus levis*, cf. *Lonchidion* sp.), heterodontiform sharks (*Heterodontus canaliculatus*), squatiniforms or orectolobiforms, sclerorhynchiforms, rajiforms (*Pseudohypolophus* sp., *Protoplatyrhina* sp.), lamniform sharks (*Hispidaspis gigas*, *Cretolamna appendiculata*, *Paraisurus* sp., *Cretodus* sp., *Protolamna* sp., *Odontaspis* sp., *Scapanorhynchus* sp., *Palaeoanacorax* sp.), chimaeriforms (*Elasmodus* sp., *Ischyodus* sp.), acipenseriforms (Polyodontidae), semionotiforms (*Lepidotes* sp.), pycnodontiforms, amiiforms, aspidorhynchiforms, pholidophoriforms, ichthyodectiforms, albuliforms, enchodontiforms, blochiid perciforms (*Cylindracanthus* sp.), cryptobranchoid salamanders (*Eoscapherpeton gracile*), frogs, plesiosaurs, macrobaenid, trionychid, carettochelyid, adocid, nanshiunghchelyid, and lindholmemyidid turtles, lizards, paralligatorid crocodyliforms, ornithopods, ankylosaurs, neoceratopsians, sauropods, non-avian theropods, birds, and eutherian mammals (Nesov 1977b, 1978, 1981a, b, 1985b, 1988a, b, 1992a, 1993, 1995, 1997; Nesov and Borkin 1983; Nesov and Golovneva 1983; Nesov and Krassovskaya 1984; Mertinene and Nesov 1985, 1991; Nesov and Mertinene 1986; Nesov and Udovichenko 1986; Glickman et al. 1987; Roček and Nesov 1993; Nesov et al. 1994; Gao and Nesov 1998; Danilov 1999; Averianov 2000, 2001, 2002b; Averianov and Archibald 2005; Averianov and Sues 2007; Danilov and Syromyatnikova 2008; Skutschas 2009; Syromyatnikova and Danilov 2009; Danilov et al. 2011; Averianov and Sues 2012a; Skutschas 2013; Sues and Averianov 2013).

Non-azhdarchid pterosaurs are represented by ornithocheirids, which are known from isolated teeth, a rostrum fragment, and some other bones (Averianov et al. 2003; Averianov 2007a, 2008).

**Paleoenvironment.** Coastal marine.

**Material.** Azhdarchidae indet: ZIN PH 61/44, edentulous jaw fragment; ZIN PH 80/44, coracoid fragment; ZIN PH 44/44, proximal manual phalanx of non-wing digit; ZIN PH 55/44, fragment of second or third wing phalanx with T-shaped cross-section.

Nesov (1990a: 8) reported a “fragment of small edentulous jaw” from Khodzhakul, which is different from ZIN PH 61/44, but cannot be presently located in the collection.

**References.** Glickman et al. 1987; Nesov 1990a, 1997; Bakhurina and Unwin 1995; Unwin et al. 1997; Unwin and Bakhurina 2000; Unwin et al. 2000; Averianov 2007b, 2010.

**Comments.** Unwin and Bakhurina (2000: fig. 21.1) misspelled the locality name as "Khodzhakuluk."

### Locality 6. Sheikhdzheili (Fig. 3)

**Geographic position.** Northern extremity of the Sheikhdzheili Range, southwestern Kyzylkum Desert, Karakalpakistan, Uzbekistan.

**Stratigraphy.** Upper part of Khodzhakul Formation.

**Age.** Early Cenomanian.

**Depositional environment.** In the sample there are some shark teeth reworked from late Albian levels (*Paraisurus* sp., *Palaeoanacorax* sp.) (Nesov and Mertinene 1986). According to these authors, teeth of some other marine sharks could also have been reworked from the lower part of the Khodzhakul Formation. The chondrichthyan taxa that were undoubtedly present in the paleoenvironment, based on preservation of their teeth, are hybodontiforms, *Scapanorhynchus*, and *Ischyodus*. The intensive reworking of older strata was caused by the marine retreat during the early Cenomanian regression. The depositional environment was likely deltaic within an estuarine basin. The dominance of gastropods rather than bivalves indicates a shallow bay with still water conditions. Among fishes the most abundant forms were those with sclerophagous dentitions (pycnodonts, *Lepidotes* sp.) which apparently consume the numerous small gastropods at this site. There are phosphatized cones of conifers (Cupressaceae) and leaves of angiosperms (Platanaceae) (Nesov 1992a, 1997).

**Associated fauna.** Crustaceans, abundant small brackish-water gastropods (*Mathildella* sp.), synechodontiforms (*Synechodus dispar*), hybodontiforms (*Hybodus hodzhakulensis*, *Hybodus nukusensis*, *Polyacrodus* spp., *Acrodus levis*), heterodontiforms (*Heterodontus canaliculatus*), squatiniforms or orectolobiforms, sclerorhynchiforms (*Ischyrhiza* sp.), rajiforms (*Protoplatyrhina* sp.), lamniforms (*Hispidaspis gigas*, *Cretodus* sp., *Protolamna* sp., *Odontaspis* sp., *Scapanorhynchus* sp., *Palaeoanacorax* sp.), chimaeriforms (*Ischyodus* sp.), semionotiforms (*Lepidotes* sp.), pycnodontiforms, amiiforms, aspidorhynchiforms, albuliforms, ichthyodectiforms, albanerpetontids, cryptobranchoid salamanders (*Eoscapherpeton gracile*), frogs, macrobaenid, carettochelyid, adocid, nanhsiungchelyid, trionychid, and lindholmemydid turtles, plesiosaurs, lizards, crocodyliforms, ankylosaurs, ornithopods, neoceratopsians, sauropods, non-avian theropods, birds, and eutherian mammals (Riabinin 1931; Nesov 1977a, 1981b, 1985b, 1988b, 1992a, 1993, 1995, 1997; Nesov and Golovneva 1983; Mertinene and Nesov 1985; Glickman et al. 1987; Nesov et al. 1989; Mertinene and Nesov 1991; Roček and Nesov 1993; Gao and Nesov 1998; Gardner and Averianov 1998; Danilov 1999; Averianov 2000, 2002b; Averianov and Archibald 2005; Averianov and Sues 2007; Skutschas 2007, 2013; Danilov and Syromyatnikova 2008; Syromyatnikova and Danilov 2009; Danilov et al. 2011; Syromyatnikova 2011; Averianov and Sues 2012a; Sues and Averianov 2013; Vitek and Danilov 2014).

Non-azhdarchid pterosaurs in the assemblage are represented by Ornithocheiridae, which are known from isolated teeth and rostrum fragments (Averianov et al. 2003; Averianov 2007a, 2008).

**Paleoenvironment.** Estuarine.

**Material.** Azhdarchidae indet.: ZIN PH 40/44, edentulous jaw fragment; ZIN PH 81/44, proximal rib fragment; ZIN PH 51/44, distal fragment of proximal manual phalanx of non-wing digit.

**References.** Nesov 1990a, 1997; Bakhurina and Unwin 1995; Unwin et al. 1997; Unwin and Bakhurina 2000; Averianov 2007b, 2008.

**Comments.** Bakhurina and Unwin (1995) noted a wing-phalanx fragment from Sheikhdzheili without a ventral ridge. This specimen may be referable to Ornithocheiridae.

### Locality 7. Dzharakuduk [=Dzhara-Khuduk, Dshyrakuduk, Bissekty] (Fig. 4)

**Geographic position.** Central Kyzylkum Desert, Navoiy Province, Uzbekistan.

**Stratigraphy.** Bissekty Formation (Nesov 1990b; Archibald et al. 1998; Nesov et al. 1998).

**Age.** Middle-late Turonian.

**Depositional environment.** The Bissekty Formation is dominated by well-sorted, medium-grained, quartz-dominated, and heavily cross-bedded sandstone with 12 laterally discontinuous intraformational conglomerates. The depositional environment has been interpreted as a braided fluvial system periodically flooded by marine waters, which deposited intraformational conglomerates (Archibald et al. 1998; Redman and Leighton 2009). Nesov intensively collected microvertebrates at Dzharakuduk sites during the period from 1977 to 1994. The microvertebrate remains accumulated on outcrop surfaces due to the wind denudation of the sand matrix and thus Nesov mostly employed surface collecting. However, the surface is contaminated by the fossils from the overlying marine Aitym Formation, notably by numerous teeth of several species of marine sharks. To explain the paradoxical coexistence in one site of marine, freshwater, and terrestrial species Nesov hypothesized that the locality was formed in a channel connecting the estuaries and freshwater basins and the mass mortality of freshwater and brackish-water organisms was caused by wind driven influx of marine or freshwater respectively (Nesov 1990a, 1997; Roček and Nesov 1993). Screen-washing of about 76 tons of freshly excavated matrix from the Bissekty Formation at Dzharakuduk from 1997 to 2006 showed that only few shark species are present in the fluvial part of the Bissekty Formation (see list below) (Archibald et al. 1998).

**Associated fauna.** Crustaceans, insects, gastropods, bivalves, hybodontiforms (*Hybodus kansaiensis*, *Polyacrodus* spp.), sclerorhynchiforms (*Ischyrhiza* sp.), rajiforms (*Myledaphus tritus*), lamniforms (*Scapanorhynchus* sp.), acipenseriforms, amiiforms, lepisosteiforms (*Atractosteus turanensis*), aspidorhynchiforms, pholidophoriforms, ichthyodectiforms (*Aidachar paludalis*), albuliforms, diverse salamanders and frogs, macrobaenid, adocid, trionychid (two taxa) and lindholmemydid turtles, lizards, crocodyliforms, ankylosaurs (*Bissektipelta archibaldi*), ornithopods (*Levnesovia transoxiana*), neoceratopsians (*Turanoceratops tardabilis*), sauropods, non-avian theropods, birds and mammals (Multituberculata, Spalacotheriidae, Deltatheridiidae, Asioryctitheria, *Paranyctoides quadrans*, Zhelestidae, Zalambdalestidae) (Riabinin 1931, 1935; Kurzanov 1976; Nesov and Trofimov 1979; Nesov and Khosatzky 1980; Nesov 1981a, b; 1982, 1984a, b, c, 1985a, b, 1986a, b, 1987a, b, 1988a, b, c, d, 1989, 1990b, 1992a, b, 1995, 1997; Nesov and Borkin 1983; Mertinene and Nesov 1985, 1991; Martinson et al. 1986; Nesov and Mertinene 1986; Nesov et al. 1989, 1998; Nesov and Yarkov 1989; Kielan-Jaworowska and Nesov 1990, 1992; Nesov and Panteleyev 1993; Roček and Nesov 1993; Brinkman et al. 1994; Currie et al. 1994; Archibald et al. 1998; Nesov and Panteleeva 1999; Panteleyev 1999; Averianov 2002a; Archibald and Averianov 2005, 2012; Danilov and Parham 2005; Szalay and Sargis 2006; Averianov and Sues 2007, 2012b; Danilov 2007; Feldmann et al. 2007; Rezvyi 2007; Skutschas 2009, 2013; Sues and Averianov 2009a, b, 2013; Syromyatnikova and Danilov 2009; Averianov et al. 2010; Chester et al. 2010; Mkhitaryan and Averianov 2011; Chester et al. 2012; Averianov and Archibald 2013a, b; Danilov and Vitek 2013; Vitek and Danilov 2013).

**Paleoenvironment.** Coastal plain.

**Material.** *Azhdarcho lancicollis*: more than 200 mostly fragmentary cranial and postcranial bones in ZIN PH and CCMGE collections.

**References.** Nesov 1984c, 1986a, 1989, 1990a, 1997; Glickman et al. 1987; Nesov et al. 1987; Bakhurina and Unwin 1995; Unwin et al. 1997; Unwin and Bakhurina 2000; Averianov 2010.

**Comments.** The bone fragment ZIN PH 183/44, identified as a preaxial carpal in Averianov (2010: fig. 29), is actually a fragment of a juvenile ulna. This bone as well as some other newly recognized bones will be described elsewhere. The Dzharakuduk locality has been mistakenly confused with the nearby locality Itemir and the locality Beleuta in Kazakhstan (Bakhurina and Unwin 1995; Unwin et al. 1997; Unwin and Bakhurina 2000; Unwin 2001).

**Figure 4.**
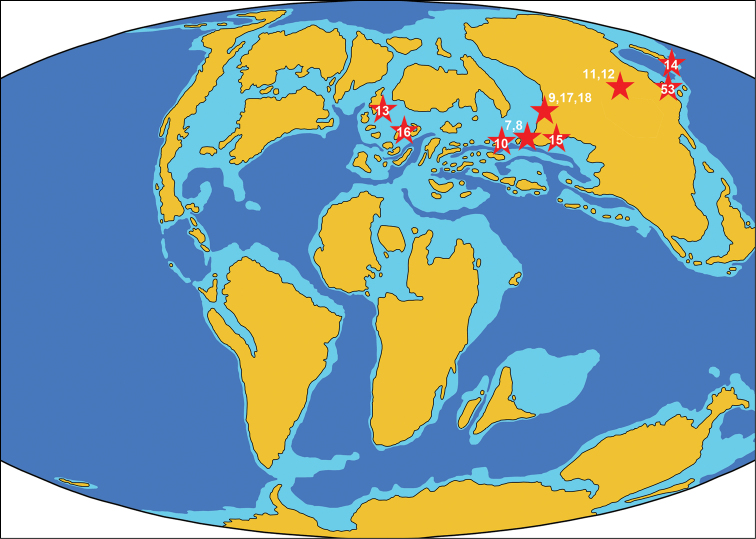
Paleogeographic map of the Late Cretaceous (90 Ma) showing Turonian-to-Santonian-age localities of azhdarchids. The numbers of localities correspond to the list in the text. The map is modified from http://jan.ucc.nau.edu/rcb7/90moll.jpg

### Locality 8. Zenge Kurgan 3 (Fig. 4).

**Geographic position.** Right bank of the Amu Darya River between the villages of Bezergen and Kulatau, Khorezm District, Uzbekistan.

**Stratigraphy.** Conglomerate of the Bissekty (?) Formation.

**Age.** Turonian.

**Depositional environment.** There are some marine elements in the fauna (*Heterodontus* sp., *Hispidaspis* sp., enchodontiforms), which are absent in the fluvial deposits of the Bissekty Formation at Dzharakuduk, northeast of Zenge Kurgan 3. These marine elements and conglomerates may indicate deposition in a submarine delta.

**Associated fauna.** Crustaceans, gastropods, bivalves, hybodontiforms (*Hybodus kansaiensis*, *Polyacrodus* spp.), heterodontiforms (*Heterodontus* sp.), sclerorhynchiforms (*Ischyrhiza* sp.), rajiforms (*Myledaphus tritus*), lamniforms (*Hispidaspis* sp., *Scapanorhynchus* sp.), acipenseriforms, amiiforms, lepisosteiforms, aspidorhynchiforms, pholidophoriforms, ichthyodectiforms, enchodontiforms, albuliforms, salamanders, frogs, adocid and trionychid turtles, lizards, crocodyliforms, ankylosaurs, ornithopods, sauropods, non-avian theropods, birds, and eutherian mammals (Danilov et al. 2011; Zelenkov and Averianov 2011).

**Paleoenvironment.** Coastal marine.

**Material.** Azhdarchidae indet.: ZIN PH 82/44, edentulous jaw fragment; ZIN PH 82/44, femoral diaphysis.

**References.** Averianov 2007b, 2008.

### Locality 9. Tyulkili [=Kankazgan] (Fig. 4)

**Geographic position.** Lower Syr-Darya Uplift, North-East Aral Sea region, Kyzylorda Province, Kazakhstan.

**Stratigraphy.** Zhirkindek Formation.

**Age.** Late Turonian – Coniacian.

**Depositional environment.** The Zhirkindek Formation at Tyulkili hills is composed of sands interbedded with clays and silts (Shilin 1998; Kordikova et al. 2001). The ferruginous sandstone at the bottom produces numerous plant remains, including 36 species of angiosperms (Shilin 1982, 1986, 1998). The next higher bed consists of yellow-grey and grey clays and represents an ingression of brackish waters in the region. It has produced fossilized wood and numerous remains of invertebrates and vertebrates (Nesov 1995, 1997). The main fossiliferous horizon at the Tyulkili locality is confined to the middle sandstone bed, about 18 m above the base of the Zhirkindek Formation (Kordikova et al. 2001). The upper part of the Zhirkindek Formation at Tyulkili hills is composed of light grey clays that have yielded abundant plant remains and a bird feather (Shilin 1986: fig. 3; Nesov 1992a).

**Associated fauna.** Crustaceans, gastropods, bivalves, brachiopods, hybodontiforms (*Hybodus* sp., *Polyacrodus* sp.), lamniforms (*Protolamna* sp., *Scapanorhynchus* sp., *Cretodus longiplicatus*), lepisosteiforms, salamanders, trionychid and lindholmemydid turtles, plesiosaurs, lizards, crocodyliforms, ankylosaurs, ornithopods, neoceratopsians, sauropods, non-avian theropods, and birds (Martinson 1990, 1997; Nesov 1995, 1997; Kordikova et al. 2001; Averianov 2007c, 2009; Averianov and Sues 2009; Skutschas 2013).

**Paleoenvironment.** Estuarine.

**Material.** Azhdarchidae indet.: ZIN PH 56/43, fragment of distal portion of ulna; ZIN PH 38/43, poorly preserved fragment of radius or ulna; ZIN PH 13/43, small fragment of the first wing phalanx (?); ZIN PH 54/43, dorsal vertebra.

**References.** Averianov 2007b, 2008.

### Locality 10. Khidzorut (Fig. 4)

**Geographic position.** Near Khidzorut village, Vayots Dzor Province, Armenia.

**Stratigraphy.** Marine sandstone.

**Age.** Late Turonian, *Subprionocyclus neptuni* ammonite zone.

**Depositional environment.** Shallow-water marine. The proximity of the coastal line is indicated by the imprint of an angiosperm leaf (*Dicotylophyllum* sp.) (Averianov and Atabekyan 2005).

**Associated fauna.** Gastropods, inoceramid and trigoniid bivalves, ammonites (*Reesidites minimus*, *Tongoboryceras rhodanicus*, *Lewesiceras mantelli*, *Scaphites geinitzi*) (Averianov and Atabekyan 2005).

**Paleoenvironment.** Coastal marine.

**Material.** Azhdarchidae indet.: CCMGE 1/12671, fragment of distal portion of ulna.

**References.** Nesov 1990a, 1997; Bakhurina and Unwin 1995; Unwin et al. 1997; Unwin and Bakhurina 2000; Averianov and Atabekyan 2005; Averianov 2007b.

**Comments.** CCMGE 1/12671 was erroneously interpreted as a radius fragment by Nesov (1997) and Averianov and Atabekyan (2005).

### Locality 11. Bayshin Tsav (Figs 4, 5)

**Geographic position.** Southern Gobi Aimag, Mongolia.

**Stratigraphy.** Upper part of Baynshire Formation.

**Age.** Late Turonian – Santonian (Averianov and Sues 2012a).

**Depositional environment.** Alternating thin layers of fining−upward units (from coarse sands to mud) intercalated with many layers of yellowish brown to reddish brown, coarse−grained sandstone and relatively fine−grained conglomerate, probably representing point-bar deposits. The bone-bearing bed is one of the bluish white, fine− to coarse−grained sandstone layers that alternate with gray mudstone layers, containing isolated dinosaur bones and teeth (Watabe et al. 2009). The section of Baynshire Formation at Bayshin Tsav has been interpreted as a cycle of alluvial-lacustrine deposits (Tsybin and Kurzanov 1979).

**Associated fauna.** Conchostracans, ostracods, hybodontiforms (*Hybodus* “*asiaticus*” [nomen nudum]), carretochelyid, trionychid, adocid, nanhsiungchelyid, and lindholmemydid turtles, ankylosaurs, ornithopods, and non-avian theropods (Shuvalov and Chkhikvadze 1975; Sukhanov and Narmandakh 1975; Chkhikvadze 1976; Maryańska 1977; Perle 1977, 1979, 1981; Shuvalov and Trusova 1979; Tsybin and Kurzanov 1979; Barsbold and Perle 1980; Barsbold 1981, 1983; Efimov 1983, 1988a, b; Tumanova 1987; Clark et al. 1994; Norman and Kurzanov 1997; Norman and Sues 2000; Storrs and Efimov 2000; Sukhanov 2000; Suzuki and Narmandakh 2004; Danilov and Syromyatnikova 2008; Zanno 2010; Danilov et al. 2011).

**Paleoenvironment.** Fluvial plain.

**Material.** Azhdarchidae indet.: MPC-Nd 100/303, cervical IV.

**References.** Watabe et al. 2009.

**Figure 5.**
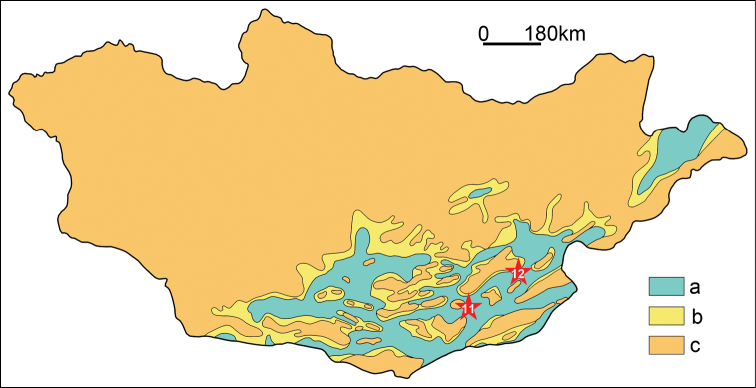
Paleogeographic map of Mongolia for the Santonian showing the Bayshin Tsav (11) and Burkhant (12) azhdarchid localities. a, lakes and lacustrine-alluvial plain; b, alluvial-proluvial plain; c, denudation area. The map was modified from Martinson (1982: fig. 6).

### Locality 12. Burkhant (Figs 4, 5)

**Geographic position.** Eastern Gobi Aimag, Mongolia.

**Stratigraphy.** Upper part of Baynshire Formation.

**Age.** Late Turonian – Santonian (Hicks et al. 1999; Averianov and Sues 2012a).

**Depositional environment.** The locality exposes reddish brown, fine− to medium−grained cross-bedded sandstone intercalated with thin layers of gray to reddish brown mudstone. The beds containing vertebrate fossils are interpreted as point-bar deposits of a meandering river (Watabe et al. 2009).

**Associated fauna.** Trionychid and adocid turtles, sauropods, non-avian theropods (Perle et al. 1999; Suzuki and Narmandakh 2004; Watabe et al. 2009; Danilov et al. 2011).

**Paleoenvironment.** Fluvial plain.

**Material.** Azhdarchidae indet.: MPC-Nd 100/302, associated atlas-axis and cervicals III and posterior fragment of cervical IV.

**References.** Watabe et al. 2009.

### Locality 13. Hope Point (Fig. 4)

**Geographic position.** Kent, England, United Kingdom.

**Stratigraphy.** St Margaret’s Member of the White Chalk Formation.

**Age.** Middle Coniacian (Martill et al. 2008).

**Depositional environment.** Marine.

**Associated fauna.** Not indicated. The White Chalk (Turonian-Maastrichtian) has yielded a rich fauna of marine invertebrates and vertebrates (Benton and Spencer 1995; Smith and Batten 2002).

**Paleoenvironment.** Coastal marine.

**Material.** Azhdarchidae indet., associated cervicals: NHMUK 16479a, cervical III; NHMUK 16479b, posterior end of cervical VI; NHMUK 16479c, unidentified bone fragment.

**References.** Martill et al. 2008.

### Locality 14. Amagami dam (Fig. 4)

**Geographic position.** Near Mifune City, Kumamoto Prefecture, Japan.

**Stratigraphy.** Middle part of the "Upper" Formation of Mifune Group.

**Age.** Coniacian-Santonian. A zircon fission-track age for the “Upper“ Formation of Mifune Group (86.4±7.8 Ma) places it at the Coniacian-Santonian boundary (Kusuhashi et al. 2008)

**Depositional environment.** The Mifune Group is subdivided into three informal formations, “basal,” “lower,” and “upper.” The “Basal“ and “Upper” formations are non-marine in origin and the Lower Formation was deposited under shallow-water marine conditions with Cenomanian-age ammonites, inoceramid and trigoniid bivalves, and sharks (Matsumoto 1939; Matsumoto and Noda 1986; Ikegami et al. 2000; Ikegami and Tomida 2003; Sha 2007; Kitamura 2013). The vertebrate remains are confined to the several levels within the "Upper" Formation (800-1000 m), which is composed of red mudstone, greenish fine-grained sandstone, and dacitic tuffaceous beds. MDM 349 was found in the middle part of the "Upper" Formation in a coarse sandstone bed, which is about 30 cm thick and has muddy patches between two tuff beds (Ikegami et al. 2000). The “Upper” Formation contains broad-leaf angiosperm megafossils (*Protophyllum* and *Populus*) and is interpreted as terrestrial, including swamp, deposits under the influence of a warm climate with alternating dry and wet seasons (Matsukawa and Obata 1994).

**Associated fauna.** Bivalves, amiiforms, lepisosteiforms, carretochelyid, trionychid, adocid, and nanhsiungchelyid turtles, crocodyliforms, ankylosaurs, ornithopods, possible ceratopsians, non-avian theropods, and eutherian mammals (Tamura 1979; Tamura et al. 1991; Matsukawa and Obata 1994; Hirayama 1998; Chure et al. 1999; Setoguchi et al. 1999; Ikegami and Tomida 2003; Matsukawa et al. 2005; Kusuhashi et al. 2008).

**Paleoenvironment.** Fluvial plain.

**Material.** Azhdarchidae indet.: MDM 349, posterior fragment of cervical IV; depositary unknown, distal end of wing metacarpal; KCM VP 000,120, proximal fragment of first wing phalanx.

**References.** Ikegami and Tamura 1996; Okazaki and Kitamura 1996; Ikegami 1997; Ikegami et al. 2000; Ikegami and Tomida 2003; Obata et al. 2007.

### Locality 15. Kansai (Fig. 4)

**Geographic position.** Near Kansai village, Northeastern Fergana Depression, Sughd Province, Tajikistan.

**Stratigraphy.** Cross-bedded red sandstone and conglomerate, upper part of the Yalovach Formation (Vyalov 1945a, b; Verzilin 1963; Rozhdestvensky 1977).

**Age.** Early Santonian (Nesov 1997). The Yalovach Formation was dated previously as late Turonian (Verzilin 1963).

**Depositional environment.** Fluvial and oxbow lake deposits according to Rozhdestvensky (1977). Rozhdestvensky excluded the possibility of submarine deltaic deposition because of the absence of benthic forms. He though that marine nektonic organisms (sharks, holosteans, some turtles) could enter rivers. Acipenserid remains indicate the presence of a large river flowing from the mountains (Nesov and Verzilin 1983). The Kansai locality was formed on a low coastal plain about 40 km east from the Fergana Gulf of Tethys (Nesov and Verzilin 1983: fig. 1). Later Nesov (1990a) thought that the locality was formed in a channel between a marine bay and a lagoon. Finally he argued that the locality was formed in a shallow brackish water basin rather than a river channel (Nesov 1997). The inference concerning an estuarine depositional environment is supported by presence of brackish-water chondrichthyans (*Baibishia baibishe*, *Protoplatyrhina* sp.), which are not found in the fluvial Cretaceous deposits of the region.

**Associated fauna.** Crustaceans, bivalves, hybodontiforms (*Hybodus kansaiensis*, *Polyacrodus* spp.), sclerorhynchiforms (*Ischyrhiza* sp.), rajiforms (*Myledaphus glickmani*, *Baibishia baibishe*, *Protoplatyrhina* sp.), acipenseriforms, amiiforms, pholidophoriforms, aspidorhynchiforms, ichthyodectiforms, albuliforms, salamanders, frogs, macrobaenid, adocid, trionychid, and lindholmemydid turtles, lizards, crocodyliforms, ankylosaurs, ornithopods, sauropods, non-avian theropods, birds, and eutherian mammals (Martinson 1965; Rozhdestvensky 1977; Nesov and Verzilin 1983; Mertinene and Nesov 1985, 1991; Nesov 1985b, 1988a, b, 1993, 1995, 1997; Nesov and Udovichenko 1986; Efimov 1988a; Nesov et al. 1994; Averianov 2000; Archibald and Averianov 2003; Alifanov and Averianov 2006; Averianov and Sues 2007, 2012a; Danilov et al. 2007, 2011; Syromyatnikova and Danilov 2009; Vitek and Danilov 2010; Averianov and Alifanov 2012; Skutschas 2013).

**Paleoenvironment.** Estuarine.

**Material.** Azhdarchidae indet.: ZIN PH 50/43, fragment of proximal end of humerus; ZIN PH 10/43, fragment of second or third wing phalanx with ventral ridge.

**References.** Nesov 1984c, 1990a, 1997; Glickman et al. 1987; Bakhurina and Unwin 1995; Unwin et al. 1997; Unwin and Bakhurina 2000; Averianov 2004, 2007b, 2008.

### Locality 16. Iharkút (Fig. 4)

**Geographic position.** Bauxite mine near the villages of Iharkút and Németbánya, Bakony Mountains, Veszprém County, Hungary.

**Stratigraphy.** Csehbánya Formation.

**Age.** Santonian.

**Depositional environment.** The Csehbánya Formation consists of channel and alluvial plain deposits, including sandstone bodies and paleosol horizons, and the bone beds probably formed in a shallow channel or pond that was episodically filled by debris flows (Ősi et al. 2005; Makádi 2013; Rabi et al. 2013). The pycnodontiform fishes and mosasauroid lizards lived in freshwater based on geochemical analysis of their remains (Kocsis et al. 2009).

**Associated fauna.** Gastropods, bivalves, freshwater ostracods, lepisosteiforms, pycnodontiforms, albanerpetontids, frogs, bothremydid turtles, lizards (scincomorphs and mosasauroids), crocodyliforms, ankylosaurs, ornithopods, neoceratopsians, non-avian theropods, and birds (Ősi 2004a, b, 2005, 2008; Ősi et al. 2005, 2007, 2010a, b, 2012a, b, 2014; Makádi 2006, 2013; Kocsis et al. 2009; Ősi and Makádi 2009; Dyke and Ősi 2010; Szentesi and Venczel 2010, 2012; Ősi and Buffetaut 2011; Makádi et al. 2012; Rabi et al. 2012, 2013; Szentesi et al. 2013).

**Paleoenvironment.** Fluvial plain.

**Material.** *Bakonydraco galaczi*: MTM V2007.110.1, mandible (holotype); MTM V2007.111.1–22, symphyseal fragments of dentary; isolated postcranial bones from Iharkút mine (MTM collection) referred originally to Azhdarchidae indet. (Ősi et al. 2005, 2011).

**References.** Ősi et al. 2005, 2011.

### Locality 17. Shakh-Shakh (Fig. 4)

**Geographic position.** Lower Syr-Darya Uplift, northeastern Aral Sea region, Kyzylorda Province, Kazakhstan.

**Stratigraphy.** Bostobe Formation.

**Age.** Santonian – early Campanian (Nesov 1997; Averianov and Sues 2012a).

**Depositional environment.** Initially considered fresh-water deposits based on the mollusk fauna (Martinson et al. 1966; Martinson and Nikitin 1978; Martinson 1990). However, the abundance and diversity of chondrichthyan fishes suggests deposition in a marine bay, an estuary where the mouth of a nearby river was separated from the sea by a bar of sediments (Nesov and Mertinene 1986; Nesov 1988a, 1990a).

**Associated fauna.** Bivalves, gastropods, hybodontiforms (*Hybodus kansaiensis*, *Polyacrodus* spp.)., rajiforms (*Myledaphus glickmani*), amiiforms, aspidorhynchiforms, ichthyodectiforms, salamanders, frogs, adocid, nanhsiungchelyid, lindholmemydid, and trionychid turtles, lizards, crocodyliforms, ornithopods, non-avian theropods, birds, and eutherian mammals (Rozhdestvensky 1964, 1968, 1970; Bazhanov 1972; Kuznetsov 1976; Martinson and Nikitin 1978; Nesov and Mertinene 1982, 1986; Suslov 1982; Kuznetsov and Shilin 1983; Mertinene and Nesov 1985; Kuznetsov and Chkhikvadze 1987; Nesov 1988a, 1992a, 1995, 1997; Nesov and Khisarova 1988; Martinson 1990, 1997; Norman and Sues 2000; Kordikova et al. 2001; Dyke and Malakhov 2004; Godefroit et al. 2004; Averianov 2007c; Danilov et al. 2007, 2011, in press; Syromyatnikova and Danilov 2009, 2013; Vitek and Danilov 2010; Averianov and Sues 2012a; Skutschas 2013; Averianov et al. in press).

**Paleoenvironment.** Coastal plain around marine bay along the shores of the Turgai Strait.

**Material.** *Aralazhdarcho bostobensis*: Anterior fragment of mid-cervical vertebra (holotype) and other isolated bones in ZIN PH collection.

**References.** Nesov 1984c, 1990a, 1997; Bakhurina and Unwin 1995; Unwin et al. 1997; Unwin and Bakhurina 2000; Averianov 2004, 2007b, 2008.

**Comments.** In the paleontological literature, the Bostobe Formation is often confused with the Beleuty [=Beleuta, =Beleutinskaya] Formation (Rozhdestvensky 1964, 1968; Godefroit et al. 2004; Weishampel et al. 2004). The Beleuty Formation was established for the Upper Cretaceous continental deposits bearing only plant remains in the Chu-Sarysu Depression, east of the Lower Syr-Darya Uplift (Nikiforova 1960), and this term has since been abandoned (Lobacheva 1979). The Beleuta Formation was also erroneously cited as the unit exposed at the Dzharakuduk locality (locality 7 in this list) in Uzbekistan (Bakhurina and Unwin 1995; Unwin and Bakhurina 2000; Unwin 2001), which, in fact, is the Bissekty Formation (former Taikarshi beds).

### Locality 18. Akkurgan (Fig. 4)

**Geographic position.** Lower Syr-Darya Uplift, northeastern Aral Sea region, Kyzylorda Province, Kazakhstan.

**Stratigraphy.** Bostobe Formation.

**Age.** Santonian – early Campanian (Nesov 1997).

**Depositional environment.** As for Shakh-Shakh locality.

**Associated fauna.** Acipenseriforms, adocid, lindholmemydid, and trionychid turtles, crocodyliforms, ornithopods, and non-avian theropods (Shilin and Suslov 1982; Nesov and Kaznyshkin 1983; Nesov 1995, 1997; Norman and Kurzanov 1997; Norman and Sues 2000; Syromyatnikova and Danilov 2009; Danilov et al. 2011; Godefroit et al. 2012).

**Paleoenvironment.** Coastal plain around marine bay along the shores of the Turgai Strait.

**Material.** *Aralazhdarcho bostobensis*: WDC Kz-001, posterior part of mandible (holotype of *Samrukia nessovi*).

**References.** Buffetaut 2011; Naish et al. 2012.

**Comments.** Naish et al. (2012) described a gigantic bird, *Samrukia nessovi*, based on a fragment of the posterior portion of the mandible from the Akkurgan locality, which was subsequently correctly reinterpreted as a pterosaur (Buffetaut 2011). It is similar to the mandible of *Quetzalcoatlus* A (Kellner and Langston 1996: fig. 4) in having a peculiar posterolateral process of the lateral cotyle of the mandibular glenoid, which is absent in ornithocheirids or pteranodontids (Wellnhofer 1985; Bennett 2001). Most likely *Samrukia nessovi* is an azhdarchid pterosaur and is possibly a subjective junior synonym of *Aralazhdarcho bostobensis*, known from the same formation at Shakh Shakh.

### Locality 19. Malaya Serdoba [Малая Сердоба] (Fig. 6)

**Geographic position.** Near Malaya Serdova village, Penza Province, Russia.

**Stratigraphy.** Rybushka Formation.

**Age.** Early Campanian (Glazunova 1972).

**Depositional environment.** The vertebrate remains are concentrated in a phosphorite conglomerate within a glauconitic sandstone (Pervushov et al. 1999). The bed was formed in shallow marine waters, as indicated by benthic invertebrates, enriched by phosphorus due to marine upwelling (Nesov 1990a).

**Associated fauna.** Marine gastropods, bivalves, scaphopods, lingulid brachiopods, diverse marine chondrichthyans, including chimaeriforms (represented by dental remains and coprolites), enchodontiforms, protostegid turtles, plesiosaurs, and mosasaurs (Sintsov 1872; Bogolyubov 1911; Tsaregradskii 1926; Rozhdestvensky 1973; Nesov 1997; Arkhangelsky 1999; Pervushov et al. 1999).

**Paleoenvironment.** Coastal marine.

**Material.** Azhdarchidae indet.: mid-cervical posterior fragment, holotype of *Bogolubovia orientalis* (nomen dubium; whereabouts unknown); ZIN PH 48/43, jaw fragment; ZIN PH 48/43, fragment of distal portion of wing metacarpal.

**References.** Bogolyubov 1914; Khozatsky and Yur'ev 1964; Glickman et al. 1987; Nesov and Yarkov 1989; Nesov 1991; Bakhurina and Unwin 1995; Unwin et al. 1997; Unwin and Bakhurina 2000; Averianov 2002b, 2007b, 2008; Averianov et al. 2005, 2008.

**Comments.** This material may belong to *Volgadraco bogolubovi*, described from locality 21 in the same formation.

**Figure 6.**
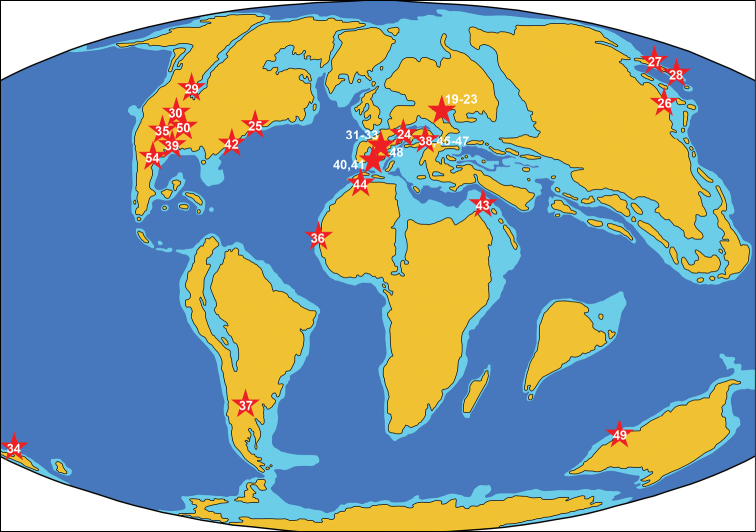
Paleogeographic map of the latest Cretaceous (65 Ma) showing Campanian-Maastrichtian-age localities of azhdarchids. The numbers of localities correspond to the list in the text. The map is modified from http://jan.ucc.nau.edu/rcb7/65moll.jpg

### Locality 20. Beloe Ozero [Белое Озеро] (Fig. 6)

**Geographic position.** Near Beloe Ozero village, Saratov Province, Russia.

**Stratigraphy.** Rybushka Formation.

**Age.** Early Campanian.

**Depositional environment.** As for locality 19. In addition to skeletal remains, there are numerous shark coprolites in the phosphorite bed.

**Associated fauna.** Brachiopods, bivalves, lingulid brachiopods, heterodontiforms (*Heterodontus* sp.), squatiniforms (*Squatina hasei*), rajiforms (*Squatirhina* sp.), lamniforms (*Cretolamna appendiculata*, *Squalicorax kaupi*, *Pseudocorax laevis*, *Archaeolamna kopingensis*, *Eostriatolamia* sp.), chimaeriforms (*Ischyodus bifurcates*, *Amylodon karamysh*, *Edaphodon* sp., *Elasmodus* sp.), enchodontiforms and other marine osteichthyans, marine turtles, plesiosaurs, and mosasaurs (Ochev 1976; Arkhangelsky et al. 2007; Averianov and Popov 2014).

**Paleoenvironment.** Coastal marine.

**Material.** Azhdarchidae indet.: ZIN PH 14/43, edentulous jaw fragment; ZIN РН 55/43, dorsal vertebra; ZIN РН 52/43 and 53/43, coracoid fragments; ZIN РН 47/43, fragment of proximal portion of first wing phalanx; ZIN РН 51/43, fragment of first(?) wing phalanx.

**References.** Averianov 2007b, 2008; Averianov and Panteleyev 2009; Averianov and Popov 2014.

**Comments.** Material may belong to *Volgadraco bogolubovi*.

### Locality 21. Shyrokii Karamysh [Широкий Карамыш] (Fig. 6)

**Geographic position.** Near Shyrokii karamysh village, Saratov Province, Russia.

**Stratigraphy.** Rybushka Formation.

**Age.** Early Campanian.

**Depositional environment.** As for locality 19.

**Associated fauna.** Bivalves, lamniforms (*Cretolamna* sp., *Eostriatolamia* sp., *Pseudocorax laevis*), chimaeriforms (*Amylodon karamysh*, *Edaphodon* sp., *Ischyodus bifurcates*), enchodontiforms, mosasaurs, plesiosaurs, and mosasaurs (Averianov and Popov 1995; Pervushov et al. 1999; Averianov et al. 2008).

**Paleoenvironment.** Coastal marine.

**Material.** *Volgadraco bogolubovi*: SGU 46/104a, rostrum fragment (holotype); SGU 47/104a, cervical III; SGU 48/104a, cervical IX; SGU 49/104a, notarium fragment; SGU 51/104a, first wing phalanx(?) fragment; SGU 50/104a, femur fragment.

**References.** Averianov 2008; Averianov et al. 2008.

### Locality 22. Saratov 2 [Саратов 2] (Fig. 6)

**Geographic position.** Saratov city, Saratov Province, Russia.

**Stratigraphy.** Pudovkino Formation.

**Age.** Early Campanian, *Belemnitella mucronata mucronata* zone(Averianov et al. 2005).

**Depositional environment.** Sandy marl with rare bioturbation traces. The Pudovkino Formation is a facies equivalent of the more sandy Rybushka Formation (Alekseev et al. 2005). It was deposited in deeper marine waters, more distant from the coastline as evident from rarity of terrestrial and nearshore marine organisms.

**Associated fauna.** Echinoids, oysters, belemnites (*Belemnitella mucronata*), mosasaurs (Pervushov et al. 1999; Averianov et al. 2005).

**Paleoenvironment.** Coastal marine.

**Material.** Azhdarchidae indet.: SGU 35/104a, fragment of distal portion of radius.

**References.** Averianov et al. 2005; Averianov 2007b, 2008.

### Locality 23. Polunino 2 [Полунино 2] (Fig. 6)

**Geographic position.** Right bank of the Volga River, Volgograd Province, Russia.

**Stratigraphy.** Unnamed unit.

**Age.** Late (?) Campanian.

**Depositional environment.** The vertebrate remains come from a phosphoritic conglomerate within the glauconitic sandstone (Pervushov et al. 1999). In this bed there are numerous shark coprolites. Invertebrate remains are rare and poorly preserved. The fossil-bearing bed was formed in shallow marine waters under upwelling conditions. The remains of crocodyliforms and possible ankylosaurs indicate proximity to land.

**Associated fauna.** Crustaceans, bivalves, various chondrichthyans and osteichthyans, including acipenseriforms, chelonioid marine turtles, plesiosaurs, mosasaurs, crocodyliforms, and ankylosaurs (?) (Nesov 1995, 1997; Averianov and Yarkov 2000, 2004a, b; Averianov 2002b).

**Paleoenvironment.** Coastal marine.

**Material.** Azhdarchidae indet.: VGI 231/4, fragment of humeral head; ZIN PH 58/43, fragment of distal portion of ulna.

**References.** Averianov and Yarkov 2004b; Averianov 2008.

**Comments.** VGI 231/4 was referred originally to Ornithocheiridae indet. based on the not saddle-shaped humeral head (Averianov and Yarkov 2004b). In all known azhdarchids the humeral head is saddle-shaped except possibly in a recently found specimen of *Aralazhdarcho bostobensis* (ZIN PH 57/43). Because of this reference of VGI 231/4 to Azhdarchidae is more likely.

### Locality 24. Muthmannsdorf (Fig. 6)

**Geographic position.** Niederösterreich, Austria.

**Stratigraphy.** Grünbach Formation, Lower Gosau Subgroup.

**Age.** Early Campanian (Sachs and Hornung 2006; Summesberger et al. 2007).

**Depositional environment.** The Gosau Group is a marginal continental to shallow marine succession of Late Cretaceous and Paleocene age, deposited in several small synclinal basins along the northern margin of the Eastern Alpine zone (Faupl et al. 1987; Wagreich and Marschalko 1995). The Lower Gosau Subgroup (upper Turonian-Campanian) is characterized by terrestrial to shallow-water marine facies associations: alluvial fan and fan delta deposits, shallow-marine sandstones and sandy limestones, and storm-influenced nearshore and shelf deposits (Wagreich and Faupl 1994). The Grünbach Formation comprises interbedded coal seams, coaly siltstones, sandstones, and conglomerates that were deposited under freshwater to nearshore marine conditions (Kvaček and Herman 2004; Sachs and Hornung 2006; Rabi et al. 2013).

**Associated fauna.** Gastropods, stem pleurodiran and cryptodiran turtles, choristoderes, lizards, crocodyliforms, ankylosaurs, and ornithopods (Bunzel 1871; Seeley 1881; Nopcsa 1926; Buffetaut 1979, 1989; Pereda Suberbiola and Galton 2001; Sachs and Hornung 2006; Rabi et al. 2013).

**Paleoenvironment.** An estuarine, brachyhaline-brackish environment based on its fauna, flora, and lithofacies (Sachs and Hornung 2006). Plant fossils indicate a subtropical climate with seasonal aridity and a paleoenvironment of coastal freshwater ponds and oxbow lakes surrounded by swampy lowlands (Kvaček and Herman 2004; Herman and Kvaček 2007).

**Material.** Azhdarchidae indet.: UWPI 2349/101, posterior mandible fragment. UWPI 2349/101, proximal portion of humerus. Unnumbered specimens, wing phalanges fragments.

**References.** Bunzel 1871; Seeley 1881; Nopcsa 1926; Wellnhofer 1980; Jianu et al. 1997; Buffetaut et al. 2011.

**Comments.** The first pterosaur fossil from Muthmannsdorf, the posterior fragment of a lower jaw, was described as the articular of a lizard (Bunzel 1871: 14 and pl.6, figs 6-7). Seeley (1881) established a new species, *Ornithocheirus buenzeli*, based on lower jaw fragment. The species epithet was spelled as “*bunzeli*” by Wellnhofer (1980), but later its spelling (Sachs and Hornung 2006: 416; Buffetaut et al. 2011: 335) was corrected to “*buenzeli*” in accordance with ICZN (1999: Article 32.5.2.1). Wellnhofer (1980) restricted *Ornithocheirus buenzeli* to the jaw fragment only and referred the postcranial remains to *Ornithocheirus* sp. Nesov (1991) cited unpublished opinion by Unwin that "*Ornithocheirus*" *buenzeli* might be an azhdarchid. Jianu et al. (1997) referred the humerus to Nyctosauridae on the basis of supposedly hatchet-shaped deltopectoral crest.

### Locality 25. Chesapeake and Delaware Canal (Fig. 6)

**Geographic position.** New Castle County, Delaware, USA.

**Stratigraphy.** Merchantville Formation.

**Age.** Early Campanian (Kennedy and Cobban 1993).

**Depositional environment.** At the northern end of its occurrence, the Merchantville Formation is mainly a sequence of thin very fine to fine-grained sandy and silty beds and, less commonly, thick beds of glauconitic sand. Discontinuous layers of rounded pale-gray siderite concretions are abundant in the thin-bedded sequence. In the west-central outcrop area, the Merchantville Formation is a thick-bedded sequence of dark-gray clayey quartz silts and dark-greenish-gray quartz-glauconite sands. In the southwest, the formation is a dark-gray massive silty fine to very fine glauconite-quartz sand. Fossil casts are abundant, and locally in the southwest, very fossiliferous siderite concretions are common in the lower part of the formation (Owens et al. 1970).

**Associated fauna.** Sponges, gastropods, bivalves, ammonites, annelids, crustaceans, hybodontiforms (*Lonchidion babulskii*), sclerorhynchiforms (*Ischyrhiza mira*), lamniforms (*Scapanorhynchus texanus*, *Squalicorax pristodontus*, *Cretolamna appendiculata*, *Odontaspis* sp.), acipenseriforms, enchodontiforms, pycnodontiforms, pelomedusid, chelonioid and trionychid turtles, mosasaurs, crocodyliforms, ornithopods, and non-avian theropods (Gaffney and Zangerl 1968; Baird and Galton 1981; Lauginiger 1984, 1988; Russell 1988; Kennedy and Cobban 1993; Hilton and Grande 2006).

**Paleoenvironment.** Coastal marine.

**Material.** Azhdarchidae indet.: YPM-PU 21820, cervical III; YPM-PU 22359, humerus fragment; YPM-PU 21821, femur and tibia fragments.

**References.** Baird and Galton 1981.

**Comments.** Bennett (1994) thought that these specimens might belong to *Pteranodon*. Referred to ?*Pteranodon* by Barrett et al. (2008). According to Howse (1986: 316) YPM-PU 21820 is not referable to *Pteranodon* because of lack of preexapophyses and it differs from *Nyctosaurus* by its much larger size.

### Locality 26. Shangpanzhen (Fig. 6)

**Geographic position.** Near Linhai City, Zhejian Province, China.

**Stratigraphy.** Middle Member of Tangshang Formation.

**Age.** Early Campanian (Mu and Cai 1992; Cai and Wei 1994).

**Depositional environment.** The Tangshang Formation (250-2600 m) consists of alternating volcanics and sandy conglomerates (Chen and Chang 1994). The Tangshang Formation in western part of Zhejian Province consists of Lower Cretaceous strata which have Albian radiometric dates (Lu et al. 2006; Jin et al. 2007). Near Linhai city a black shale occurs within the upper part of the conglomeratic suite and contains specimens of the teleostean fish *Paraclupea* and the conchostracan *Linhaiella* (Chen and Chang 1994). At the pterosaur locality the Tangshang Formation is composed of lacustrine sediments interbedded with calcareous tuffs (Unwin and Lü 1997). The locality was formed in a large fresh or brackish-water inland lake (Chen 2000: figs 3, 4).

**Associated fauna.** The non-avian theropods and dinosaur eggs reported from the Tangshang Formation apparently come from the Early Cretaceous part of the formation (Dong 1979; Mateer 1989). At the pterosaur locality, only a complete skeleton of a small theropod, possible a dromaeosaurid, has been found (Unwin and Lü 1997).

**Paleoenvironment.** Lacustrine.

**Material.** *Zhejiangopterus linhaiensis*: ZMNH M1330, relatively complete skull (holotype; ZMNH M1324, skull and cervicals; ZMNH M1325; relatively complete skeleton lacking the skull; ZMNH M1323, relatively complete skeleton; ZMNH M1328, relatively complete skeleton; ZMNH M1329, partial skeleton.

**References.** Cai and Wei 1994; Unwin and Lü 1997.

### Locality 27. Enbetsu [=Embetsu] (Fig. 6)

**Geographic position.** Hokkaidō Prefecture, Japan.

**Stratigraphy.** Hakobuchi Formation or Group.

**Age.** Late Campanian.

**Depositional environment.** The local stratigraphy of the Cretaceous deposits on Hokkaido is very complex due to local tectonics and the stratigraphic nomenclature differs considerably from author to author (Ando et al. 2001, 2010). According to Shigeta et al. (2010), the Hakobuchi Formation consists mainly of sandstone and is divided into the four lithological units (in ascending order): IVa, sandstone in association with conglomerate, sandy mudstone and coal beds; IVb, sandy mudstone; IVc, sandstone with intercalations of conglomerate and sandy mudstone beds; and IVd, sandy mudstone. Based on ammonites, unit IVa is late Campanian and IVb is early Maastrichtian in age. The pterosaur fossil was found with the ammonite *Metaplacenticeras subtilistriatum* and thus come from unit IVa of the Hakobuchi Formation (Chitoku 1996; Shigeta et al. 2010: fig. 6). The Hakobuchi Formation in the Nakagawa area, close to Enbetsu town, consists mainly of sandy shallow-water marine facies, which suggests a storm-dominated shore face to shelf environment. Fluvial and estuarine sediments are limited to the western sections, indicating the paralic to fluvial nature of the sediments occasionally recorded as incised valley fills (Ando et al. 2010).

**Associated fauna** (combined list for the late Campanian – early Maastrichtian levels of the Hakobuchi Formation). Inoceramid bivalves, ammonites, dermochelyid and cheloniid turtles, plesiosaurs, and mosasaurs (Hirayama and Chitoku 1996; Sato et al. 2012).

**Paleoenvironment.** Coastal marine. The pollen from the coal beds is dominated by angiosperms and indicates a subtropical climate (Sato 1961; Miki 1977).

**Material.** Azhdarchidae indet.: HMG 1052, cervical III.

**References.** Chitoku 1996; Obata et al. 2007.

**Comments.** Referred to ?Ornithocheiridae by Barrett et al. (2008). Not referable to the Ornithocheiroidea because of small lateral pneumatic foramen.

### Locality 28. Awaji Island (Fig. 6)

**Geographic position.** Hyōgo Prefecture, Japan.

**Stratigraphy.** Seidan Formation, Izumi Group.

**Age.** Late Campanian. *Pachydiscus awajiensis* Zone (Saegusa and Furutani 2004).

**Depositional environment.** The Campanian–Maastrichtian Izumi Group is distributed in a narrow, long area along Shikoku, Awaji Island and the Kii Peninsula to the south-west. This group was likely deposited in a strike-slip basin and exhibits an upward-deepening sequence from a nearshore to slope basin, which is dominated by turbidites. The Izumi Group of Awaji Island is divided, in ascending order, into the Seidan, Anaga, Kita-ama, Nada and Shimonada formations (Morozumi 1985). The Seidan Formation is composed of mudstones, sandstones, and alternating beds of sandstone and mudstone (Misaki et al. 2014).

**Associated fauna.** Bivalves, ammonites, trionychid turtles, and mosasaurs (Morozumi 1985; Tanimoto 2005, 2010; Sato et al. 2012; Misaki et al. 2014).

**Paleoenvironment.** Coastal marine.

**Material.** Azhdarchidae indet., depository unknown, posterior fragment of cervical (postzygapophyseal width 6.5 cm).

**References.** Saegusa and Furutani 2004; Tanimoto 2010.

**Comments.** A wing-metacarpal shaft fragment UMUT MM 7978 from the lower Maastrichtian Kita-ama Formation of Awaji Island, referred to ?Azhdarchidae (Obata et al. 2007), may indeed belong to this group but this cannot be firmly established.

### Locality 29. Dinosaur Provincial Park (Figs 6, 7)

**Geographic position.** Near Brooks, Alberta, Canada.

**Stratigraphy.** Dinosaur Park Formation.

**Age.** Late Campanian.

**Depositional environment.** The Dinosaur Park Formation (70 m) is divided into lower sandy zone, middle muddy zone, and upper Lethbridge Coal Zone (Eberth 2005). The lower zone consists primarily of fine- to medium-grained, cross-bedded sandstones and was deposited in fluvial-channel environments. The middle zone consists primarily of massive to laminated, organic-rich mudstones with abundant root traces, and thin beds of bentonite. It was deposited in overbank and floodplain environments. The upper zone consists of several seams of low-rank coal interbedded with mudstones and siltstones. The Dinosaur Park Formation was deposited on an alluvial plain near the coast of the Bearpaw Sea, a large inland sea that was part of the Western Interior Seaway. There are two paleoecological assemblages in the formation: inland in the lower zone and coastal in the middle zone (Brinkman 1990; Brinkman et al. 2005b). These two communities and the lithology of the Dinosaur Park Formation reflect a transgression of the Bearpaw Sea, which culminated in the deposition of marine shales of Bearpaw Formation above the Dinosaur Park Formation (Eberth 2005). The Lethbridge Coal Zone represents transitional terrestrial-marine environment, a tidally influenced estuary, with marine chondrichthyans and mosasaurs (Beavan and Russell 1999; Brinkman et al. 2005a; Caldwell 2005). The coastal community is characterized by a diverse chondrichthyan fauna, whereas in the freshwater fluvial beds only three chondrichthyan taxa are present (Brinkman et al. 2005b; Neuman and Brinkman 2005). Unfortunately, there is no published information on the stratigraphic positions for occurrences of pterosaur bones in the Dinosaur Park Formation.

**Associated fauna.** Freshwater gastropods and bivalves, hybodontiforms (*Hybodus montanensis*), orectolobiforms, rajiforms (*Myledaphus tritus*), acipenseriforms, aspidorhynchiforms, lepisosteiforms, amiiforms, other intermediate holosteans (Holostean A and B), elopomorphs, osteoglossomorphs, hiodontiforms, albuliforms, esociformes, other intermediate teleosts, albanerpetontids, salamanders, frogs, baenid, macrobaenid, chelydrid, adocid, nanhsiungchelyid, and trionychid turtles, plesiosaurs, choristoderes, lizards, crocodyliforms, ankylosaurs, pachycephalosaurs, ceratopsians, ornithopods, non-avian theropods, birds, and mammals (Currie and Koppelhus 2005; Fox and Naylor 2006; Hilton and Grande 2006; Longrich 2006, 2008, 2009; Evans and Reisz 2007; Evans et al. 2007, 2009; Newbrey et al. 2007; Arbour et al. 2009; Longrich and Currie 2009; Matsumoto and Evans 2010; Ryan et al. 2012; Averianov and Archibald 2013b; Gardner and DeMar 2013; Larson and Currie 2013; Wilson et al. 2013).

**Paleoenvironment.** Fluvial coastal plain and estuarian (Eberth and Brinkman 1997; Eberth 2005).

**Material.** Azhdarchidae indet.: RTMP 92.83, associated skeleton of an immature individual including cervical IV, rib, humerus, pteroid, metacarpals III and IV, and tibia; RTMP 89.36.254, cervical IV; RTMP 96.12.369, juvenile cervical V; RTMP 81.16.107, cervical V anterior fragment; RTMP 81.16.182, scapulocoracoid fragment; RTMP 1991.36.374, fragmentary humerus; RTMP 80.16.651, fragment of proximal portion of humerus; RTMP 82.16.303 and 97.12.163, fragments of distal portions of humerus; RTMP 65.14.398, ulna; RTMP 80.16.1367, fragmentary ulna, originally described as a femur (Currie and Russell 1982); RTMP 85.36.211, fragment of distal portion of metacarpal III; RTMP 87.36.16, wing metacarpal; RTMP 72.1.1 and 82.19.295, fragments of proximal portions of first wing phalanx; RTMP 88.36.92, proximal portion of femur fragment; RTMP 91.36.616, distal portion of femur; RTMP 92.83.6, metatarsal III or IV.

**References.** Russell 1972; Currie and Russell 1982; Currie and Jacobsen 1995; Godfrey and Currie 2005; Sullivan and Fowler 2011.

**Comments.** Godfrey and Currie (2005) distinguished two forms of azhdarchids from the Dinosaur Park Formation: a smaller form, similar in size to *Montanazhdarcho minor*, and a larger one, similar to *Quetzalcoatlus* sp. Two smaller fragments of humeri (RTMP 82.16.303 and 97.12.163) have fully ossified distal ends whereas in specimens about twice as large (RTMP 92.83.4 and 1991.36.374) the distal ends are poorly ossified. Actually, the distal epiphysis may not be fused to the shaft in the figured specimen RTMP 92.83.4 (Godfrey and Currie 2005: fig. 16.6C-E). The presence of two forms may well be explained by sexual dimorphism or interspecies variation within two closely related species. The two known fourth metacarpals (RTMP 87.36.16 and 92.83.1) have "normal" proportions, not markedly shortened as in the holotype of *Montanazhdarcho minor*. I see no reason to infer the presence of *Montanazhdarcho* in the Dinosaur Park Formation.

Godfrey and Currie (2005) cited also another, non-azhdarchid pterosaur in the Dinosaur Park Formation. This identification is based on two similar specimens, RTMP 79.14.247 and 88.50.1. The former specimen was interpreted as the distal end of a pterodactyloid tibia (Currie and Padian 1983) or distal end of a wing metacarpal (McGowen et al. 2002). However, most recently this specimen was reinterpreted as an avian tibiotarsus (Buffetaut 2010). Currently there is no evidence for the presence of non-azhdarchid pterosaurs in the Dinosaur Park Formation.

**Figure 7.**
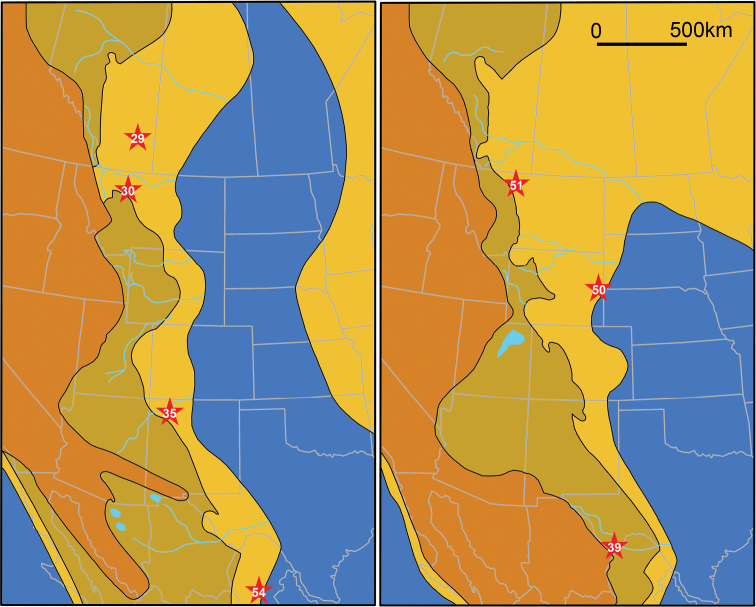
Paleogeographic map of middle North America for late Campanian (left) and early Maastrichtian (right) showing localities of azhdarchids. The numbers of localities correspond to the list in the text. The maps are modified from http://energy.cr.usgs.gov/coal_poster/cretcoals

### Locality 30. Egg Mountain (Figs 6, 7)

**Geographic position.** Near Choteau, Montana, USA.

**Stratigraphy.** Upper Two Medicine Formation (Padian 1984).

**Age.** Late Campanian (Rogers et al. 1993).

**Depositional environment.** The upper part of the Two Medicine Formation was deposited during the final stages of the Bearpaw transgression (Horner et al. 1992). It contains a lacustrine varve-like sequence associated with a pulmonate-dominated gastropod fauna. The climate was characterized by sporadic or possibly seasonal precipitation (Varricchio 1993). The pterosaur remains occurred in a well-laminated freshwater limestone (Padian 1984). The bones were associated with stromatolites and unionid freshwater bivalves and embedded in a limestone concretion.

**Associated fauna.** Bivalves, lizards, ornithopods, non-avian theropods, and mammals (Horner and Makela 1979; Horner 1982; Padian 1984; Montellano 1988; Montellano et al. 2000; Varricchio et al. 2002; DeMar et al. 2012).

This large azhdarchid possible coexisted with the non-azhdarchid azhdarchoid *Montanazhdarcho minor*, which was found at an undisclosed locality in the Two Medicine Formation in Glacier County, Montana, USA (Padian et al. 1995; McGowen et al. 2002).

**Paleoenvironment.** Coastal plain. According to Padian (1984), the region at the time of deposition was part of a "proximal lowland" about 700 km from the mid-continental seaway. According to the paleogeographic map used here (Fig. 7a) this distance is only about 270 km.

**Material.** Azhdarchidae indet.: YPM-PU 22446, associated fragmentary humerus, radius, and complete distal syncarpal.

**References.** Padian 1984; Padian and Smith 1992.

### Locality 31. Sainte-Foy and Massecaps (Fig. 6)

**Geographic position.** Near Cruzy village, Hérault, Languedoc-Roussillon, France.

**Stratigraphy.** Grès à Reptiles Formation.

**Age.** Late Campanian – early Maastrichtian.

**Depositional environment.** The non-marine Late Cretaceous in Saint-Chinian area of southern France is represented by red beds consisting of conglomerates, sandstones, and clays and by freshwater limestones, known as the “Grès à Reptiles”, overlain by basal Cenozoic red clays (Buffetaut 2005). Vertebrate remains occur, sometimes in abundance, in all these facies, the most productive localities being in the clay layers.

**Associated fauna.** Gastropods, bivalves, coelacanthiforms, lepisosteiforms, albanerpetontids, frogs, bothremydid and solemydid turtles, lizards, crocodyliforms, ankylosaurs, ornithopods, sauropods, non-avian theropods, birds, and eutherian mammals (Buffetaut 1998, 2001; Buffetaut et al. 1999; Cavin et al. 2005; Martin and Buffetaut 2005; Walker et al. 2007; Díez Díaz et al. 2013).

**Paleoenvironment.** Fluvial plain.

**Material.** Azhdarchidae indet.: MC SF69, anterior cervical fragment. MC M3929, distal syncarpal. Unnumbered, crushed wing metacarpal.

**References.** Buffetaut 2001, 2008.

### Locality 32. Bastide Neuve (Fig. 6)

**Geographic position.** Near Fox-Amphoux village, Var, Provence-Alpes-Côte d'Azur, France.

**Stratigraphy.** Grès à Reptiles Formation.

**Age.** Late Campanian – early Maastrichtian.

**Depositional environment.** The vertebrate-bearing beds of the Grès à Reptiles Formation in the region consist of clays and sandstone lenses. The bones atthe Bastide-Neuve locality come from yellowish sandy clays (Buffetaut et al. 2006).

**Associated fauna.** Hybodontiforms, lepisosteiforms, bothremydid turtles, crocodyliforms, ankylosaurs, ornithopods, sauropods, non-avian theropods and birds (Buffetaut et al. 1995, 2000, 2006; Le Loeuff and Buffetaut 1998; Tong et al. 1998).

**Paleoenvironment.** Fluvial plain.

**Material.** Azhdarchidae indet.: poorly preserved humerus in private collection.

**References.** Buffetaut et al. 2006; Buffetaut 2008.

### Locality 33. Laño (Fig. 6).

**Geographic position.** Near Victoria city, Basque Country, Spain.

**Stratigraphy.** Unnamed Formation.

**Age.** Late Campanian.

**Depositional environment.** The Cretaceous deposits at the Laño quarry consist of the lower alluvial system, lower palustrine system, upper alluvial system, and the lower coastal system (Astibia et al. 1991, 1999). The lower coastal system contains teeth of marine reptiles and sharks, including the myliobatiform *Rhombodus binkhorsti*. The remains of terrestrial vertebrates are confined to three associated fossiliferous beds (known as L1A, L1B and L2) at the bottom of the lower alluvial system. The sedimentary structures of these beds are consistent with channel areas within an extensive braided river (Pereda Suberbiola et al. 2000).

**Associated fauna.** Lepisosteiforms, elopomorphs(?), albanerpetontids, salamanders, frogs, dortokid, bothremydid, pelumedosid, and solemydid turtles, lizards, snakes, crocodyliforms, ankylosaurs, ornithopods, sauropods, non-avian theropods, and eutherian mammals (Astibia et al. 1991, 1999; Gheerbrant and Astibia 1994, 2012).

**Paleoenvironment.** Coastal plain.

**Material.** Azhdarchidae indet: MCNA 8563, edentulous jaw fragment; MCNA 8563, cervical VI; MCNA 8564, notarium; also other mostly undescribed cervicals, wing bones, sacrum, and femur.

**References.** Astibia et al. 1991; Buffetaut 1999; Pereda Suberbiola et al. 2007.

### Locality 34. Mangahouanga Stream (Fig. 6)

**Geographic position.** North Island, New Zealand.

**Stratigraphy.** Conglomeratic facies of the Maungataniwha Member of the Tahora Formation (Molnar and Wiffen 1994).

**Age.** Campanian (Isaac et al. 1991) or late Campanian – early Maastrichtian (Vajda and Raine 2010).

**Depositional environment.** The fossils come from the boulders composed of hard, grey, medium-grained calcareous sandstone (Keyes 1977; Wiffen 1980). Except macrofossils, the boulders contain terrestrial pollen and spores and also marine dinoflagellate cysts in some samples (Vajda and Raine 2010). The boulders occur as concretions within the thick sandstone sequence exposed in the stream banks, which is of identical composition to the boulders, but slightly less calcareous. The fossiliferous beds were deposited on the continental shelf, apparently under shallow-water nearshore conditions (Wiffen and Molnar 1988).

**Associated fauna.** Mollusks, sclerorhynchiforms (*Onchopristis dunklei*), elopiforms (*Pachyrhizodus caninus*), actinopterygians of uncertain affinity (*Aethocephalichthys hyainarhinos*), protostegid turtles, plesiosaurs, mosasaurs, ankylosaurs, ornithopods, sauropods, non-avian theropods (Keyes 1977; Wiffen 1980, 1981, 1983; Wiffen and Moisley 1986; Molnar and Wiffen 1994; Fielitz et al. 1999).

**Paleoenvironment.** Coastal marine. The palynoflora indicates a mixed local vegetation dominated by podocarp conifers and angiosperms with a significant tree-fern subcanopy. The presence of taxa with modern temperate distributions, such as *Nothofagus*, Proteaceae and Cyatheaceae, indicates a mild-temperate climate and lack of severe winters (Vajda and Raine 2010).

**Material.** Azhdarchidae indet: NZGS CD 547, coracoid fragment (originally identified as a scapula); NZGS CD 467, fragment of distal portion of ulna.

**References.** Wiffen 1986; Wiffen and Molnar 1988, 1994.

### Locality 35. SMP locality 281 (=Denver’s Blowout) (Figs 6, 7)

**Geographic position.** Ah-shi-sle-pah Wilderness Study Area, San Juan Basin, New Mexico, USA.

**Stratigraphy.** Hunter Wash Member, Kirtland Formation.

**Age.** Late Campanian.

**Depositional environment.** The Hunter Wash Member is composed of mudstone/siltstone, sandstone, and coal layers (Lucas and Sullivan 2000). Dinosaur bones and fossil logs occur in the upper siltstone bed.

**Associated fauna.** Amiiformes, bothremydid, pleurosternid, baenid, adocid, nanhsiungchelyid and trionychid turtles, ankylosaurs, pachycephalosaurs, ceratopsians, and non-avian theropods (Williamson and Carr 2002; Gaffney et al. 2006; Sullivan 2006; Carr and Williamson 2010; Sullivan et al. 2011a, b, 2013).

**Paleoenvironment.** Coastal plain.

**Material.** Azhdarchidae indet.: SMP VP-1445, first wing phalanx missing distal end (holotype of *Navajodactylus boerei*). SMP locality 288: SMP VP-1853, shaft of humerus (originally identified as ulna fragment).

**References.** Sullivan and Fowler 2011.

### Locality 36. Paki (Fig. 6)

**Geographic position.** Near Dakar city, Thiès, Senegal.

**Stratigraphy.** Paki Formation (Cuny et al. 2012).

**Age.** Late Campanian; latest Campanian based on the foraminiferan *Globotruncanita* aff. *Globotruncanita calcarata* (Cuny et al. 2012).

**Depositional environment.** The Paki Formation includes a set of grey siltstones (12.50 m) and yellow siltstone (0.30 m) topped by a bed of calcareous sandstone (0.20 m), which is strongly bioturbated and silty (Cuny et al. 2012). The pterosaur fossils come from this bed together with abundant angiosperm fruits, internal molds of bivalves, indeterminate brachiopods, and fish teeth (Monteillet and Lappartient 1981; Cuny et al. 2012). The Paki Formation has also yielded a rich microfauna with benthic and planktonic foraminiferans.

**Associated fauna.** Benthic and planktonic foraminiferans, bivalves, brachiopods, and myliobatiform (*Rhombodus* sp.) (Cuny et al. 2012).

**Paleoenvironment.** Coastal marine.

**Material.** Azhdarchidae indet.: depository unknown, cervical V; long bone fragment, possibly tibia.

**References.** Lappartient and Monteillet 1980; Monteillet et al. 1982.

**Comments.** The cervical was originally described as an internal cast (Monteillet et al. 1982). According to Buffetaut (2004: 574), however, it is a cast of the outer surface.

### Locality 37. Cerro de Guerra (Fig. 6)

**Geographic position.** Río Negro Province, Argentina.

**Stratigraphy.** Allen Formation.

**Age.** Late Campanian – early Maastrichtian (Leanza et al. 2004).

**Depositional environment.** The Allen Formation is composed mainly of a red or yellowish lower sandy member, a middle lacustrine section with grey-greenish shales and an upper part with gypsum, limestones and stromatolitic limestones (Leanza et al. 2004). It was deposited during a major marine transgressive phase occurring in the Late Cretaceous (Cazau and Uliana 1972; Uliana and Dellape 1981). Facies vary from intertidal environments in the eastern part of basin to tide-dominated estuarine in the western part (Gasparini et al. 2003; Gómez et al. 2008).

**Associated fauna.** The Allen Formation has yielded some invertebrates (gastropods) and a rich fauna of vertebrates including diverse chondrichthyan and osteichthyan fishes, frogs, chelid turtles, elasmosaurid plesiosaurs, rhynchocephalians, snakes, ornithopods, ankylosaurs, sauropods, non-avian theropods, birds, and mammals (Brito 1997; Clarke and Chiappe 2001; Coria 2001; Gasparini et al. 2003; Leanza et al. 2004; Martinelli and Forasiepi 2004; Apesteguía et al. 2007; Gómez et al. 2008; González Riga et al. 2008; Novas et al. 2009; Rougier et al. 2009; Bogan et al. 2010; Juárez Valieri et al. 2010; Agnolin and Novas 2012; Agnolin et al. 2012; Apesteguía and Jones 2012; Coria et al. 2012; Currie and Carabajal 2012).

**Paleoenvironment.** Coastal marine. The paleoenvironment of the Allen Formation has been interpreted as a nearshore to restricted-marine setting (Uliana and Dellape 1981). The locality was situated on the shore of an epicontinental sea covering most of the Patagonia during the Late Cretaceous (Apesteguía and Jones 2012: fig. 4C). The fish fauna includes variety of freshwater forms (dipnoans, amiids, lepisosteids, siluriforms, and possible perciforms) as well as euryhaline taxa (batoids, aspidorhynchids) (Brito 1997; Martinelli and Forasiepi 2004; Apesteguía et al. 2007; Bogan et al. 2010). The presence of plesiosaurs (Gasparini et al. 2003) suggests at least an estuarine environment. MPCN-PV 0054 was collected from a horizon with fish vertebrae and scales, chelid shells, and elasmosaurid remains, indicating a nearshore marine depositional environment for this layer (Novas et al. 2012).

**Material.** *Aerotitan sudamericanus*: MPCN-PV 0054, anterior rostrum fragment.

**References.** Novas et al. 2012.

### Locality 38. Petreşti-Arini (Fig. 6).

**Geographic position.** Transylvanian basin, Transylvania, Romania.

**Stratigraphy.** Layer 0, top of the Bozeş Formation (Csiki-Sava et al. 2012; Vremir et al. 2013).

**Age.** Latest Campanian – earliest Maastrichtian (Vremir et al. 2013).

**Depositional environment.** Transitional marine-brackish sequence. The pterosaur wing phalanx was found in the brackish estuarian facies (Vremir 2010; Csiki-Sava et al. 2012).

**Associated fauna.** Dinosaurs (Csiki-Sava et al. 2012).

**Paleoenvironment.** Estuarine.

**Material.** *Hatzegopteryx thambema*: wing phalanx fragment.

**References.** Vremir 2010; Csiki-Sava et al. 2012; Vremir et al. 2013.

### Locality 39. Big Bend National Park (Figs 6, 7)

**Geographic position.** Southwestern Texas, USA.

**Stratigraphy.** Javelina Formation of Tornillo Group (or Javelina Member of Tornillo Formation).

**Age.** Usually considered late or «middle»-late Maastrichtian (Hunt and Lehman 2008). The tuff bed below the two *Quetzalcoatlus* sites has isotopic dates of 69.0±0.9 Ma (Lehman et al. 2006), which is early Maastrichtian. Because of the division of the Maastrichtian into two substages and the early/late substage boundary set at about 66 Ma (Gradstein et al. 2004), an early Maastrichtian age is accepted here for the *Quetzalcoatlus* localities.

**Depositional environment.** The Javelina Formation consists of well-indurated conglomeratic fluvial sandstones and variegated mudstone intervals with paleosol horizons. These strata were deposited in fluvial flood-plain and associated lacustrine environments more than 400 km (Lawson 1975b) or several hundred km (Wick and Lehman 2013) inland from the Late Cretaceous shoreline. According to the paleogeographic map used here (Fig. 7b) this distance is about 290 km. Pterosaur bones occur in several concentrations, which are very closely spaced stratigraphically (Kellner and Langston 1996). These bone concentrations are within shallow alkaline-lake deposits in abandoned stream channels. One such concentration, comprising some 235 disarticulated but closely associated to randomly scattered bones of at least 9 individuals, represents a non-attritional mass mortality, and suggests possibly gregarious behavior among these pterosaurs. The enclosing lacustrine facies lacks other vertebrate fossils, but contains charophytes, gastropods, bivalves, and arthropod trace fossils (Lehman and Langston 1996). Lawson (1975b) noted a close association of pterosaur and sauropod remains.

**Associated fauna.** Gastropods, bivalves, fishes, turtles, crocodyliforms, ankylosaurs, ceratopsids, sauropods (*Alamosaurus sanjuanensis*), and non-avian theropods (Lawson 1976; Lehman and Langston 1996; Lehman and Coulson 2002; Hunt and Lehman 2008). The Javelina Formation has also yielded wood with insect boring interpreted as termite nests (Rohr et al. 1986). The mammals reported from the Javelina Formation come from the Paleocene part of the section (Kielan-Jaworowska et al. 2004).

Q. *northropi* possible coexisted with another pterosaur, known from the partial skeleton (TMM 42489), the skull of which was figured by Wellnhofer (1991: 144). This specimen was attributed to Tapejaridae (Kellner 2004), *Tupuxuara* (Martill and Naish 2006), Thalassodromidae (Elgin and Frey 2011), or Azhdarchidae (Andres and Myers 2013). This short-faced pterosaur with shorter cervical vertebrae was found much lower in the Javelina Formation (Kellner and Langston 1996: 230).

**Paleoenvironment.** Lacustrine. The land adjacent to the lake was vegetated with palms, whereas the floodplains supported a tropical forest dominated by *Javelinoxylon* and araucarian conifers. The climate was warm, dry, and non-seasonal, with mean annual temperatures exceeding 20° and rainfall less than 1000 mm per year (Lehman and Langston 1996). *Quetzalcoatlus northropi* was a member of southern *Alamosaurus* tetrapod community (Lehman 1987, 2001; Vavrek and Larsson 2010).

**Material.** *Quetzalcoatlus northropi*: TMM 41540-3, wing skeleton (humerus, partial radius, ulna, proximal and distal syncarpals, wing metacarpal, first and second wing phalanges); numerous disarticulated bones and partial skeletons usually referred to *Quetzalcoatlus* sp.

**References.** Lawson 1975a, b; Langston 1981; Kellner and Langston 1994, 1996; Lehman and Langston 1996; Andres and Myers 2013.

**Comments.** The smaller specimens come from the Amaral site (Kellner and Langston 1996). The type locality for *Quetzalcoatlus northropi* is separated from this site by 40 km (Lawson 1975b).

### Locality 40. Chera (Fig. 6)

**Geographic position.** Near Valencia, Valencia Province, Spain.

**Stratigraphy.** Sierra Perenchiza Formation.

**Age.** Middle-late Campanian. The vertebrate-bearing beds of Chera contain the charophyte *Peckichara pectinata*, which is a biostratigraphic marker for the middle to late Campanian (Company et al. 2005).

**Depositional environment.** The Sierra Perenchiza Formation represents the beginning of the continental sedimentation, which took place in shallow, lacustrine basins during the final Cretaceous marine regression (Company et al. 2005). It consists mainly of interbedded carbonate marls and lacustrine limestones, interpreted as deposits of small ephemeral lakes and ponds of a coastal environment (Company and Szentesi 2012). These sediments were periodically exposed and subjected to pedogenic modification, developing swampy regressive sequences.

**Associated fauna.** Ostracods, gastropods, bivalves, lepisosteiforms, albanerpetontids, frogs, bothremydid, dortokid, and solemydid turtles, lizards, crocodyliforms, ankylosaurs, ornithopods, sauropods, and non-avian theropods (Company et al. 1999a, 2005, 2009a, c; Company and Szentesi 2012).

**Paleoenvironment.** Lacustrine.

**Material.** Azhdarchidae indet.: depository unknown, two wing phalanges.

**References.** Pereda Suberbiola et al. 2007.

### Locality 41. La Solana (Fig. 6)

**Geographic position.** Near Valencia, Valencia Province, Spain.

**Stratigraphy.** Upper beds of the Sierra Perenchiza Formation.

Age. Late Maastrichtian (Company et al. 2001).

**Depositional environment.** The deposits represent a lacustrine succession, composed of dark red and grey clays and silts with interbedded beds of silty marls (Company et al. 1999b).

**Associated fauna.** Ostracods, gastropods, bivalves, actinopterygians, albanerpetontids, frogs, turtles, crocodyliforms, and ornithopods (Company et al. 1998, 1999b, 2009b; Pereda Suberbiola et al. 2009).

**Paleoenvironment.** Lacustrine or a swamp environment (Company et al. 1998).

**Material.** Azhdarchidae indet.: MGUV 2271, fragment of posterior portion of cervical IV; other fragmentary cervicals and miscellaneous limb fragments in MGUV and MPV collections.

**References.** Company et al. 1999b, 2001; Pereda Suberbiola et al. 2007.

### Locality 42. Maple Hill (Fig. 6)

**Geographic position.** Pender County, North Carolina, USA.

**Stratigraphy.** Rocky Point Member of the Peedee Formation.

**Age.** Late Maastrichtian (Christopher and Prowell 2002).

**Depositional environment.** The Peedee Formation consists of dark greenish to gray, micaceous, glauconitic massive sands. The upper part of this formation has been divided into two members, the lower Rocky Point and the upper Island Creek (Harris and Self-Trail 2006). The Rocky Point Member is composed of well-cemented sandy molluscan-mold grainstone to calcareous cemented quartz arenite to unconsolidated quartz sand. The pterosaur specimen came from the bed of calcareous glauconitic arenite (Parris et al. 2004).

**Associated fauna.** Bryozoans, gastropods, bivalves, ammonites, belemnites, crustaceans, sea urchins, enchodontiforms, cheloniid turtles, plesiosaurs, crocodyliforms, mosasaurs (Parris et al. 2004). Surprisingly no sharks have been reported from the pterosaur locality although they are abundant at other sites in the Peedee Formation (Case 1979).

**Paleoenvironment.** Coastal marine.

**Material.** NJSM 18772, fragmentary femur.

**References.** Parris et al. 2004.

### Locality 43. Ruseifa (Fig. 6)

**Geographic position.** Near Amman, Jordan.

**Stratigraphy.** Formerly referred to as Phosphorite unit of Balqa [=Belqa] Group (Frey and Martill 1996). This unit has been referred also to the Amman Formation (Abed 1989; Abed and Amireh 1999) or Alhisa Formation (Pufahl et al. 2003).

**Age.** Late Maastrichtian. The ammonite *Libycoceras ismaeli* is a key fossil of the *Sphenodiscus* zone (Amard 1996).

**Depositional environment.** Coastal marine. The phosphorite unit contains four main phosphate seams at Ruseifa; the pterosaur bones most likely come from a lower seam (Frey and Martill 1996). The pelletal phosphates are rich in fragmentary bones and teeth. Beds above and below the phosphate horizon contain abundant bivalves and gastropods and indicate water depths of only a few meters (Frey and Martill 1996)

**Associated fauna.** Ammonites (*Libycoceras ismaeli*, *Didymoceras* sp.), orectolobiforms, lamniforms (*Squalicorax* sp., *Scapanorhynchus* sp., and others), enchodontiforms, tetraodontiforms, alepisauriforms, cheloniid turtles, plesiosaurs, mosasaurs, crocodyliforms, ornithopods (Avnimelech 1949; Arambourg 1954, 1959; Arambourg et al. 1959; Frey and Martill 1996; Martill et al. 1996; Mustafa and Zalmout 1999; Cappetta et al. 2000; Bardet and Pereda Suberbiola 2002).

**Paleoenvironment.** Coastal marine.

**Material.** *Arambourgiania philadelphiae*: UJA VF-1, cervical V missing its posterior end (holotype); SMNK PAL 1286, proximal fragment of first wing phalanx; SMNK PAL 1287, distal fragment of first(?) wing phalanx.

**References.** Arambourg 1954, 1959; Frey and Martill 1996; Steel et al. 1997; Martill et al. 1998.

**Comments.** A partial skeleton possibly referable to *Arambourgiania philadelphiae* has been found recently at a new Maastrichtian locality in Jordan (Wilson and Zalmout 2006). The two endocranial casts from the upper Campanian Mishash Formation of Israel referred to *Arambourgiania* sp. (Lewy et al. 1992) possibly belong to birds.

### Locality 44. Sidi Daoui (Fig. 6)

**Geographic position.** Near Khouribga, central Morocco.

**Stratigraphy.** Upper “Couche III” (Pereda Suberbiola et al. 2003).

**Age.** Late Maastrichtian (Cappetta 1987; Pereda Suberbiola et al. 2003).

**Depositional environment.** Fish vertebrae, shark and teleost teeth, and mosasaur vertebrae and teeth were found in the matrix around the skeleton and indicate marine depositional environment (Pereda Suberbiola et al. 2003). It is also confirmed by the analysis of the whole fauna (Arambourg 1952). Except for the pterosaur, the only record of a terrestrial animal found in this locality is a partial skeleton of a titanosauriform sauropod, a possible remnant of a floating carcass that drifted over a distance from a land area (Pereda Suberbiola et al. 2004).

**Associated fauna.** Marine chondrichthyan and osteichthyan fishes, bothremydid turtles, plesiosaurs, mosasaurs, sauropods (Arambourg 1952; Cappetta 1987; Noubhani and Cappetta 1997; Pereda Suberbiola et al. 2003, 2004; Bardet et al. 2004).

**Paleoenvironment.** Coastal marine. Highly productive upwelling waters indicating by intensive deposition of phosphates and very abundant and diverse remains of fishes and other marine vertebrates.

**Material.** *Phosphatodraco mauritanicus*: OCP DEK/GE 111, associated cervicals V-IX and an indeterminate bone.

**References.** Pereda Suberbiola et al. 2003.

### Locality 45. Sebeş-Glod and Râpa Roşie (Fig. 6)

**Geographic position.** Transylvanian basin, Transylvania, Romania.

**Stratigraphy.** Lower to middle part (Sebeş-Glod) and upper part (Râpa Roşie) of Sebeş Formation (Vremir et al. 2013).

**Age.** Early Maastrichtian (Sebeş-Glod) and late Maastrichtian (Râpa Roşie) (Vremir et al. 2013).

**Depositional environment.** The Sebeş Formation succession is dominated by coarse, mainly cross-bedded channel fills (gravels, sandy gravels, cross-laminated sandstones) with occasional interbedding by finer-grained red or brownish-red overbank and floodplain associations (fine laminated sandstones, silty claystones, massive mudstones), all of which was formed by a high-sinuosity fluvial system (Brusatte et al. 2013). Deposition took place under various conditions, from proximal alluvial fans to the medium and distal facies of meandering, occasionally braided, fluvial systems that exhibit local evidence for well-developed lacustrine, forested-swampy, short evaporitic stages and extensive pedogenized floodplain deposits. The vertebrate fossils originate mainly from the red overbank deposits (Vremir et al. 2013).

**Associated fauna.** Stem cryptodiran and pleurodiran turtles, crocodyliforms, ornithopods, sauropods, non-avian theropods, and birds (Csiki et al. 2010; Vremir 2010; Brusatte et al. 2013; Vremir et al. 2013).

**Paleoenvironment.** Fluvial plain.

**Material.** Sebeş-Glod: *Eurazhdarcho langendorfensis*: EME VP 312, partial skeleton.

Râpa Roşie: *Hatzegopteryx thambema*: cervical III, fragmentary coracoid, and proximal syncarpal.

**References.** Vremir 2010; Vremir et al. 2013.

**Comments.** As was noted previously, *Eurazhdarcho langendorfensis* is likely a junior subjective synonym of *Hatzegopteryx thambema*.

### Locality 46. Pui (Fig. 6).

**Geographic position.** Northwestern Haţeg Basin, Transylvania, Romania.

**Stratigraphy.** Sînpetru Formation.

**Age.** Maastrichtian.

**Depositional environment.** Braided-river-dominated alluvial system (Grigorescu et al. 1999).

**Associated fauna.** Chondrostean, holostean, and teleostean fishes, albanerpetontids, anuranans, stem cryptodiran turtles, lizards, crocodyliforms, ornithopods, sauropods, theropods, and multituberculates (Grigorescu et al. 1999).

**Paleoenvironment.** Fluvial plain.

**Material.** *Hatzegopteryx thambema*: cervical, scapula, fragment of ?humerus.

**References.** Vremir et al. 2011, 2013.

### Locality 47. Vǎlioara, Tuştea, Boiţa, and Vadu (Fig. 6).

**Geographic position.** Northwestern Haţeg Basin, Transylvania, Romania.

**Stratigraphy.** Chocolate-colored (Vǎlioara) or red (Tuştea) siltstones, upper part of the Middle Member of the Densuş-Ciula Formation (Buffetaut et al. 2003).

**Age.** Late Maastrichtian (Buffetaut et al. 2003) or early or early late Maastrichtian (Vremir et al. 2013).

**Depositional environment.** The Lower Member of the Densuş-Ciula Formation is mostly lacustrine but strongly influenced by volcanic eruptions to the west; a volcanic overprint is also present in the fluvial/lacustrine Middle Member, whereas the Upper Member comprises clastic sediments (Grigorescu 1992; Grigorescu et al. 1999).

**Associated fauna.** Gastropods, holostean and teleostean fishes, albanerpetontids, anuranans, basal cryptodiran turtles, lizards, crocodyliforms, sauropods (skeletal remains and eggs), theropods, and multituberculates (Grigorescu et al. 1990, 1994, 1999; Weishampel et al. 1991; Buffetaut et al. 2003).

**Paleoenvironment.** Fluvial plain.

**Material.** *Hatzegopteryx thambema*: Vǎlioara: FGGUB R 1083, associated skull fragments and humerus. Unnumbered specimen, fragment of mandibular symphysis.

Tuştea: FGGUB R.1625, femur.

Boiţa: wing phalanx fragment.

Vadu: cervical, scapulocoracoid.

**References.** Buffetaut and Ouaja 2002, 2003; Vremir et al. 2011, 2013.

**Comments.** The first pterosaur bones at Sînpetru and Vǎlioara were collected by Baron Franz Nopcsa at the turn of the nineteenth and twentieth centuries (see Buffetaut et al. 2003). Jianu et al. (1997) referred these specimens to the Sânpetru Formation and mentioned additional pterosaur material (two notaria, humerus, and femur) from the “same locality,” not explaining which locality they meant. These bones were referred to Pteranodontidae because of fused notarium with supraneural plate and humerus with “warped” deltopectoral crest and a caudally (ventrally in flight position) directed ulnar crest. Bennett (1989: 675, fig. 2(6, 7)) introduced “ulnar crest directed posteriorly” as a synapomorphy of Pteranodontidae based on comparison with USNM 13804, the holotype of *Bennettazhia oregonensis*, where the ulnar crest is actually missing. In azhdarchoids, the ulnar crest has the same direction (ventral or posterior depending on bone orientation) as in pteranodontids and azhdarchids. The identification of “warped” deltopectoral crest is dubious because Jianu et al. (1997) gave the same determination for the deltopectoral crest in *Cretornis hlavaci*, which is not “warped” at all. The fused notarium is present in all large pterodactyloids. Thus the bones mentioned but not described by Jianu et al. (1997) may well belong to an azhdarchid. This is in agreement with the reported absence of pneumatic foramen on posterior side of the humerus (Jianu et al. 1997).

### Locality 48. Mérigon (Fig. 6)

**Geographic position.** Ariège, Midi-Pyrénées, France.

**Stratigraphy.** Marnes d'Auzas Formation.

**Age.** Late Maastrichtian (López-Martinez et al. 2001).

**Depositional environment.** The fossil locality is in the uppermost sequence of the Marnes d’Auzas Formation, a freshwater to brackish deposit with some marine intercalations (Bilotte and Ségura 1991). ME1 04 was found at the base of a thick bed of coarse sandstone overlying a bed of sandy clay (Buffetaut et al. 1997).

**Associated fauna.** Turtles, crocodyliforms, and ornithopods (Le Loeuff et al. 1994; Buffetaut et al. 1997; Buffetaut 2008).

**Paleoenvironment.** Estuary or bay.

**Material.** Azhdarchidae indet.: ME1 04, fragmentary cervical V (estimated length ~55 cm).

**References.** Buffetaut et al. 1997; Buffetaut 2008.

**Comments.** Estimated wing-span ~9 m (Buffetaut 2008).

### Locality 49. Toothawarra Creek (Fig. 6).

**Geographic position.** Giralia Range, Western Australia, Australia.

**Stratigraphy.** Miria Formation.

**Age.** Late Maastrichtian.

**Depositional environment.** The Miria Formation occurs as a thin unit in the Cretaceous succession of the Giralia Anticline, which forms the Giralia Range (Henderson and McNamara 1985). Its deposition started with a late Maastrichtian marine transgression (Bennett and Long 1991). It consists of a cream-colored calcarenite (0.6–2 m) with abundant phosphatic grains and nodules. The fossils are usually preserved as phosphatic molds.

**Associated fauna.** Foraminiferans, sponges, corals, bryozoans, gastropods, bivalves, nautiloids, ammonites, brachiopods, echinoids, chondrichthyans, mosasaurs, and possible non-avian theropods (Henderson and McNamara 1985; Bennett and Long 1991; Long 1992; Kear et al. 2005).

**Paleoenvironment.** Coastal marine.

**Material.** Azhdarchidae indet: WAM 60.57, proximal portion of ulna.

**References.** Bennett and Long 1991.

### Locality 50. Lull 2 quarry (UCMP locality V-5620) (Figs 6, 7)

**Geographic position.** Niobrara County, Wyoming, USA.

**Stratigraphy.** Near the top of the Lance Formation (Estes 1964).

**Age.** Latest Maastrichtian (Longrich et al. 2012a).

**Depositional environment.** The Lance Formation represents a mixture of non-marine rocks (Connor 1992). Typically sandstones of intermediate thickness, somewhat regularly spaced or concentrated, occur in lower part. There are a few thin beds of coal. Relatively silt-free sandstones at the microvertebrate sites indicate a smooth, constant current, building marginal sandbars in the relatively clear waters (Estes 1964).

**Associated fauna.** Gastropods, bivalves, hybodontiforms (*Lonchidion selachios*), orectolobiforms (*Restesia americana*), sclerorhynchiforms (*Ischyrhiza avonicola*), rajiformes (*Myledaphus bipartitus*), acipenseriforms, amiiforms, lepisosteiforms, aspidorhynchiforms, elopiforms, albuliforms, esociformes, perciforms, albanerpetontids, frogs, salamanders, baenid, nanhsiungchelyid, and trionychid turtles, lizards, snakes, crocodyliforms, ornithopods, pachycephalosaurs, non-avian theropods, birds, and mammals (McKenna 1961; Brodkorb 1963; Clemens 1963, 1966, 1973; Estes 1964, 1965, 1969; Estes et al. 1969; Gaffney 1972; Novacek and Clemens 1977; Breithaupt 1982; Carpenter 1982; Estes and Sanchíz 1982; Krause 1992; Wilson et al. 1992; Gao and Fox 1996; Elzanowski et al. 2000; Gardner 2000; Holroyd and Hutchison 2002; Hope 2002; Case et al. 2005; Sankey 2008; Lyson and Joyce 2009; Elzanowski and Stidham 2011; Longrich et al. 2012a, b; Gardner and DeMar 2013).

**Paleoenvironment.** Coastal plain.

**Material.** Azhdarchidae indet.: UCMP 114286: cervical V-VI (Henderson and Peterson 2006: 195) or cervical V (Averianov 2010: 287) lacking posterior end.

**References.** Estes 1964.

**Comments.** Estes (1964: 145) referred this specimen to Pterosauria with reservation and thought that it might represent a “coccygeal structure.” Lawson (1975b) first noted that it is a cervical vertebra similar to those of *Quetzalcoatlus*. This specimen was subsequently referred to *Azhdarcho* sp. (Nesov 1984c) or Azhdarchidae indet. (Averianov 2010).

### Locality 51. Burpee Museum locality K-12 (Fig. 7)

**Geographic position.** Carter County, Montana, USA.

**Stratigraphy.** Hell Creek Formation.

**Age.** Latest Maastrichtian.

**Depositional environment.** Strata exposed at the collecting locality preserve a fining upward sequence of sediments. The basal unit is a thick, poorly sorted, and weakly cross-bedded sandstone. BMR P2002.2 was found near the top of this sandstone. Occasional specimens of angiosperm leaves were the only other fossils encountered in this sandstone unit, which is thought to represent point bar deposits. The sandstone is overlain by a clay-ball conglomerate, which is, in turn, overlain by laminated clays containing abundant remains of aquatic plants. The sequence of strata preserved at the fossil localiy is interpreted as representing a stream avulsion and subsequent development of an oxbow lake (Henderson and Peterson 2006).

**Associated fauna.** Non-avian theropods (Henderson and Peterson 2006).

**Paleoenvironment.** Coastal plain.

**Material.** Azhdarchidae indet.: BMR P2002.2, cervical V.

**References.** Henderson and Peterson 2006.

**Comments.** The specimen was originally identified as cf. *Quetzalcoatlus* sp. (Henderson and Peterson 2006).

### Review of localities of footprints possible referable to AzhdarchidaeLocality 52. Gain (Fig. 2)

**Geographic position.** Changseon Island, South Gyeongsang Province, South Korea.

**Stratigraphy.** Middle part of Haman Formation of the Hayang Group.

**Age.** Aptian-Albian.

**Depositional environment.** The Haman Formation is mainly composed of reddish shale, sandy shale, and white to greenish and gray sandstones with minor intercalated tuffaceous and pebbly sandstone. The pterosaur and dinosaur track-bearing sandstone occurs in the middle part of the Haman Formation, which consists of centimeter-scale, rhythmic alternations of fine-grained siliciclastic sediments. The vertebrate footprints and invertebrate trace fossils are accompanied with the ripple marks, mud cracks, and raindrop imprints which indicate a lakeshore environment (Kim et al. 2012).

**Associated fauna.** Invertebrate trace fossils, footprints of non-avian dinosaurs and web-footed birds (Kim et al. 2006, 2008, 2012).

**Paleoenvironment.** Lacustrine.

**Material.** *Haenamichnus gainensis*: footprints.

**References.** Kim et al. 2012.

**Comments.** *Haenamichnus* footprints are found together with *Pteraichnus*-like footprints, which are not referable to Azhdarchidae (Kim et al. 2006).

### Locality 53. Uhangri (Fig. 4)

**Geographic position.** Jeollanam Province, South Korea.

**Stratigraphy.** Upper part of the Uhangri Formation of the Haenam Group.

**Age.** Santonian - early Campanian (Hwang et al. 2002; Kim et al. 2003).

**Depositional environment.** The Uhangri Formation comprises a clastic fluviolacustrine sequence with minor volcaniclastic deposits (Chun and Chough 1995). Vertebrate tracks were found at three different levels in the upper part of the Uhangri Formation. Pterosaur tracks, associated with numerous bird and dinosaur tracks and ripple marks, were only found in the lowest track level, which consists of a well-laminated black shale. This part of the formation represents the shallow margin of a lake (Hwang et al. 2002).

**Associated fauna.** Ostracods, invertebrate trace fossils attributed to arthropods, footprints of non-avian dinosaurs and web-footed birds (Lockley et al. 1997; Yang et al. 1997; Hwang et al. 2002).

**Paleoenvironment.** Lacustrine.

**Material.** *Haenamichnus uhangriensis* and *Haenamichnus* sp.: footprints.

**References.** Lockley et al. 1997; Hwang et al. 2002.

### Locality 54. El Pelillal (Figs 6, 7)

**Geographic position.** Coahuila Province, Mexico.

**Stratigraphy.** Cerro del Pueblo Formation.

**Age.** Latest Campanian.

**Depositional environment.** The tracksite is located stratigraphically high in the sequence of the Cerro del Pueblo Formation in a reddish to light brown, fine-grained, intensely bioturbated and ripple-marked sandstone (Rodriguez-De La Rosa 2003), which may document a freshwater depositional environment with possible tidal influence (Rodriguez-De La Rosa and Cevallos-Ferriz 1998).

**Associated fauna.** Bivalves and other invertebrates, lepisosteiforms, amiiforms, trionychid, chelydrid, and kinosternoid turtles, crocodyliforms, ankylosaurs, ornithopods, ceratopsians, non-avian theropods, and birds (known from footprints) (Rodriguez-De La Rosa and Cevallos-Ferriz 1998; Eberth et al. 2004; Gates et al. 2007; Loewen et al. 2010).

**Paleoenvironment.** Lacustrine.

**Material.** *Pteraichnus* sp.: footprints.

**References.** Rodriguez-De La Rosa 2003.

**Comments.** Possible belongs to *Haenamichnus* according to Witton and Naish (2008). A bone fragment of Pterodactyloidea indet. has been reported from this site (Rodriguez-De La Rosa and Cevallos-Ferriz 1998).

### Cenomanian-Turonian extinction event and post-Turonian Pterosauria

The Cenomanian-Turonian mass extinction occurred during the peak of a global greenhouse interval, when atmospheric CO_2_ succeeded the present level at least four times (Kauffman 1995; Eaton et al. 1997; Harries and Little 1999; Benson et al. 2013). During this interval the sea level was nearly 300 m above the present stand. The principal groups affected by this mass extinction were planktonic dinoflagellates, foraminiferans, sponges, rudists, ammonoids, malacostracans, ostracodes, echinoids, bony fishes and ichthyosaurs (Sepkoski 1982, 1986; Benton 1989; Bardet 1994; Fischer et al. 2014). Approximately 28% of marine invertebrate genera became extinct during this short-term (less than 1 my) extinction event (Sepkoski 1986). Widespread extinction particularly affected tropical reef ecosystems (Kauffman 1995).

Toothed pterosaurs apparently did not survive the Cenomanian-Turonian mass extinction. Ornithocheiridae is not known after the Cenomanian and Lonchodectidae after the Turonian (Unwin 2001; Barrett et al. 2008; Andres and Myers 2013; Rodrigues and Kellner 2013). Milner (2002) reported ornithocheirids from the Santonian of England but this record is based on isolated vertebrae whose attribution to Ornithocheiridae is problematic. From the Turonian onward, all identifiable pterosaur remains belong to toothless groups (Pteranodontidae, Nyctosauridae, and Azhdarchoidea). There are a number of post-Cenomanian localities with fragmentary pterosaur bones that cannot be positively identified but possibly belong to Azhdarchidae (Table 2).

**Table 2.** Post-Cenomanian localities of Pterodactyloidea indet. which may belong to Azhdarchidae.

Locality	Geography	Stratigraphy	Age	Environment	References
Futalognko	Neuquén, Argentina	Portezuelo Fm	Turonian – early Coniacian	Fluvial	Kellner et al. 2006
Samatazawa	Hokkaidō, Japan	Upper Yezo Gr	Coniacian-Santonian	Marine	Obata et al. 1972; Sato et al. 2012
Baibishe	Kyzylorda, Kazakhstan	Bostobe Fm	Santonian – early Campanian	Estuarine	Averianov 2008
Buroinak	Kyzylorda, Kazakhstan	Bostobe Fm	Santonian – early Campanian	Fluvial	Averianov 2008
Polunino 1	Volgograd, Russia		Early Maastrichtian	Marine	Averianov 2008
Awaji Island	Hyōgo, Japan	Kita-ama Fm, Izumi Gr	Early Maastrichtian	Marine	Obata et al. 2007
Bexen	Aude, France	Marnes Rouges de Roquelongue Fm	Late Maastrichtian	Lacustrine or lagoonal	Buffetaut et al. 1996

### Other putative records of skeletal remains of Azhdarchidae

Sayão and Kellner (2001) reported on elongated cervical vertebrae from the Kimmeridgian-Tithonian Tendaguru beds in Tanzania and referred them to Azhdarchidae. However, the lack of illustrations or detailed descriptions of these specimens does not allow assessment of this identification. Elongated cervical vertebrae are also present in Ctenochasmatidae and non-azhdarchid Azhdarchoidea.

Howse (1986) referred *Doratorhynchus validus*, represented by a long mid-cervical from the Berriasian Durlstone Formation of England (Seeley 1875, 1901), to Azhdarchidae. This referral was accepted by some other authors (Wellnhofer 1991; Padian and Smith 1992; Kellner 2003). The taxonomy of pterodactyloids from the Purbeck beds is confusing and was partially revised by Howse and Milner (1995). In my opinion, all specimens described in the latter paper can be safely referred to a single taxon of Ctenochasmatidae, *Gnathosaurus macrurus* (including *Doratorhynchus validus* and *Plataleorhynchus streptophorodon*). Bakhurina and Unwin (1995: 230) mistakenly claimed that Nesov also included “*Doratorhynchus*” in Azhdarchidae. He in fact explicitly stated that “*Doratorhynchus*” does not belong to Azhdarchidae because of presence of a pneumatic foramen on the lateral side of the vertebra (Nesov 1991: 19). This mid-cervical (Martill et al. 2013: fig. 11B) cannot be attributed to Azhdarchidae also because of its continuous neural spine, which is disrupted in the middle and confined to the anterior and posterior ends in mid-cervicals of Azhdarchidae. There are also no reasons to attribute a wing metacarpal from this locality to Azhdarchidae (Martill et al. 2013: figs 4, 5). It can be referred to *Gnathosaurus macrurus*.

The fragmentary humerus PMOL AP00018 from the Aptian Doushan Formation of the Qingshan Group at Laiyang City, Shandong Province, China, considered as the stratigraphically oldest record of Azhdarchidae (Zhou 2010c), most likely belongs to Dsungaripteridae, known previously from this locality (Young 1958, 1964). It was identified as Azhdarchidae indet. based on a single character, the swollen terminal expansion of the deltopectoral crest (Zhou 2010c), which is an ontogenetic trait not particularly diagnostic for Azhdarchidae. In other aspects of its structure, this specimen does not differ from the humerus of *Dsungaripterus*.

Undescribed skeletal remains of Azhdarchidae have been reported from Aptian Elrhaz Formation in Ténéré Desert of central Niger (Sereno et al. 1998) and the Maastrichtian of Jordan (Wilson and Zalmout 2006).

An isolated vertebra from the Maastrichtian Lapurr sandstone of Kenya, identified originally as a posterior cervical of ?Azhdarchidae (O'Connor et al. 2011), is a caudal vertebra of a mosasaur.

Hone et al. (2012) reported on heavily digested pterosaur bones within a rib cage of the dromaeosaurid theropod *Velociraptor mongoliensis* from the Djadokhta Formation at Tugrikin Shireh, Mongolia. These bones were referred to Pterosauria because of thin bony walls and to Azhdarchidae because 1) this pterosaur group dominated in the Late Cretaceous; 2) azhdarchid are known from the Baynshire Formation (Watabe et al. 2009); and 3) azhdarchids “likely favoured terrestrial environments” (Hone et al. 2012: 29). This interpretation of the fossils is a good example of circular reasoning: first, they were referred to azhdarchids because these animals were terrestrial and then this finding was used to support terrestrial habits of azhdarchids (Witton and Naish In press). These bones, however, have no morphological features that would allow referral to Azhdarchidae. Furthermore, I even doubt the pterosaurian nature of these bones. The bones could have been digested to such an extent that only a thin cortical bone layer remained.

### Paleoenvironments of Azhdarchidae

In a previous most recent review of azhdarchid distribution (Witton and Naish 2008), 32 localities of these pterosaurs have been listed. From this list two localities are actually duplicates: the Oldman Formation and the Dinosaur Park Formation in Alberta, Canada. The first find (Currie and Russell 1982) came from the Judithian part of the Oldman Formation, which has been subsequently separated as the Dinosaur Park Formation (Eberth and Hamblin 1993). Similarly, the Ksar es Souk and Kem Kem region of Witton and Naish (2008) are considered here as a single locality named Taouz. The Two Medicine locality for *Montanazhdarcho minor* and the Glen Rose locality for *Radiodactylus langstoni* are excluded here because these taxa are not azhdarchids. The records from the Bexen [=Montplasir] (Buffetaut et al. 1996), Illd Formation, Upper Yezo Group (Obata et al. 1972), and Kita-ama Formation, Izumi Group (Obata et al. 2007) are excluded because the pterosaur material is too fragmentary and cannot be confidently attributed to Azhdarchidae. The Portezuelo Formation is excluded because the material cannot be determined beyond Azhdarchoidea indet. (Kellner et al. 2006; Novas et al. 2012). Thus the revised list of azhdarchid localities provided by Witton and Naish (2008) includes only 24 localities. Here I present a much more extensive list of azhdarchid localities, including 51 localities with skeletal remains and three localities with tracks.

**Figure 8.**
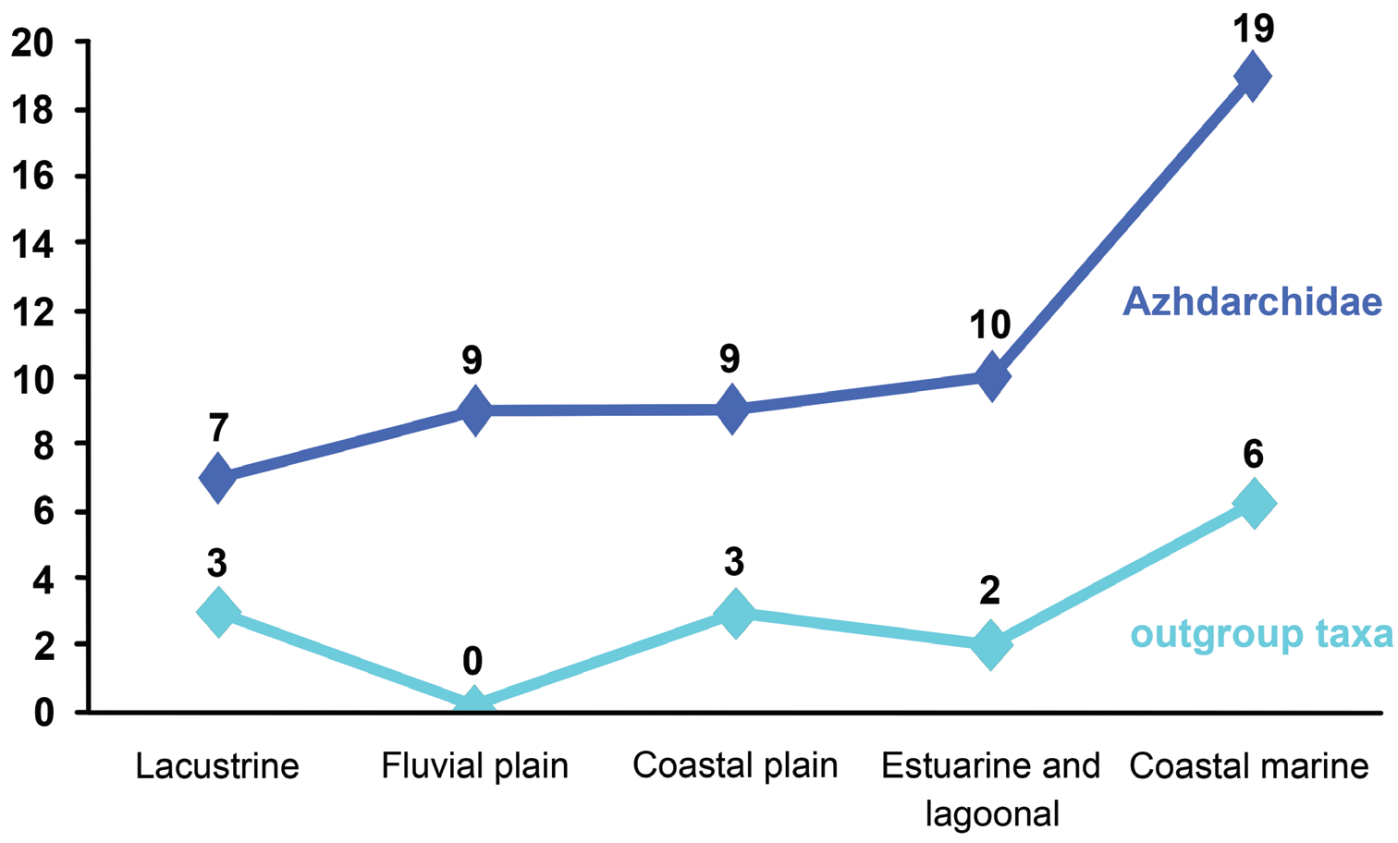
Number of azhdarchid and possible outgroup taxa localities plotted on the paleoenvironments.

According to Witton and Naish (2008: 3) “most azhdarchids are found in continental fluvial deposits […] a condition perhaps best demonstrated by the occurrence of *Quetzalcoatlus* 400 km from the nearest contemporary shoreline.” However, as was discussed above (locality 39) *Quetzalcoatlus* remains come from the lacustrine deposits only ~170 km from the nearest shoreline. In Asia, the two Santonian-age localities, Bayshin Tsav and Burkhant, are located well within the Asian landmass, but these localities are confined to prominent system of lakes that were possibly connected to Tethys (Figs 4 and 5). Other azhdarchid inland localities are usually placed very close to the contemporaneous coastlines. Based on the data presented here, 13% of azhdarchid occurrences are from lacustrine deposits, 17% from fluvial-plain deposits, 17% from coastal plain deposits, 18% from estuarine and lagoonal deposits, and 35% from coastal marine deposits (Fig. 8). There is a distinct trend of increase of azhdarchid occurrences from inland to coastal marine paleoenvironments. This trend cannot be explained only by taphonomic reasons (Witton and Naish in press), because fragile pterosaur bones could not be transported for long distances from the inhabited areas. If there was transport of pterosaur bones from more inland “terrestrial” environments to coastal marine facies, why are the latter facies not full of dinosaur bones? Dinosaur bones are generally more solid, and dinosaurs were undoubtedly more numerous than pterosaurs. In the aforementioned facies pterosaurs are more frequently found than dinosaurs. Azhdarchids likely inhabited a variety of environments, but were abundant near large continental water bodies (lakes and rivers) and most common in nearshore marine paleoenvironments, as along with the majority of other pterodactyloids.
